# Yap is required for ependymal integrity and is suppressed in LPA-induced hydrocephalus

**DOI:** 10.1038/ncomms10329

**Published:** 2016-01-12

**Authors:** Raehee Park, Uk Yeol Moon, Jun Young Park, Lucinda J. Hughes, Randy L. Johnson, Seo-Hee Cho, Seonhee Kim

**Affiliations:** 1 Shriners Hospitals Pediatrics Research Center, Temple University Lewis Katz School of Medicine, Philadelphia, Pennsylvania 19140, USA; 2 Department of Anatomy and Cell Biology, Temple University School of Medicine, Philadelphia, Pennsylvania 19140, USA; 3 Graduate Program of Biomedical Sciences, Temple University School of Medicine, Philadelphia, Pennsylvania 19140, USA; 4 Department of Cancer Biology, MD Anderson Cancer Research Center, University of Texas, Houston, Texas 77030, USA

## Abstract

Timely generation and normal maturation of ependymal cells along the aqueduct are critical for preventing physical blockage between the third and fourth ventricles and the development of fetal non-communicating hydrocephalus. Our study identifies Yap, the downstream effector of the evolutionarily conserved Hippo pathway, as a central regulator for generating developmentally controlled ependymal cells along the ventricular lining of the aqueduct. Yap function is necessary for proper proliferation of progenitors and apical attachment of ependymal precursor cells. Importantly, an injury signal initiated by lysophosphatidic acid (LPA), an upstream regulator of Yap that can cause fetal haemorrhagic hydrocephalus, deregulates Yap in the developing aqueduct. LPA exposure leads to the loss of N-cadherin concentrations at the apical endfeet, which can be partially restored by forced Yap expression and more efficiently by phosphomimetic Yap. These results reveal a novel function of Yap in retaining tissue junctions during normal development and after fetal brain injury.

## Abstract (web summary)

Failure of the ependymal cell layer that lines the ventricular surfaces of the brain can result in fetal hydrocephalus. Park *et al*. reveal a role for the Hippo pathway gene Yap in the generation of ependymal cells, and show that suppression of Yap is observed in a model of hydrocephalus.

## Graphical abstract

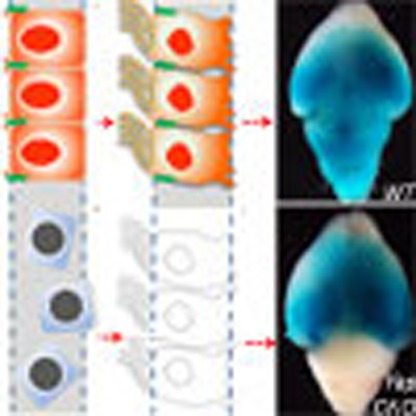

Hydrocephalus is one of the most common neurodevelopmental defects and occurs in 1–3 out of 1,000 live births. It is characterized by enlargement of cerebrospinal fluid (CSF)-filled intracerebral ventricles, causing severe mental retardation and motor dysfunction^1^,^2^. Known causes of hydrocephalus include infection, brain trauma and genetic mutation. Hydrocephalus is classified into communicating and non-communicating forms, based on presence or absence of structural blockage of CSF flow^3^,^4^,^5^. Disrupted structural integrity of the ventricular system can cause non-communicating hydrocephalus; excessive secretion of CSF from the choroid plexus, inefficient reabsorption of CSF by the subarachnoid villi and defective flow of CSF cause communicating hydrocephalus^6^.

Ependymal cells, derived from neuroepithelium, line the ventricular surface and are closely associated with hydrocephalus due to cilia defects^7^. More critically, failure of normal generation, maturation and integrity of ependymal cells can cause early onset fetal hydrocephalus through aqueductal stenosis, which blocks CSF in the narrow passage between the third and fourth ventricles^3^,^8^. Although genetic studies of hydrocephalus have demonstrated the significance of genes involving adhesion and cytoskeletal organization^7^,^9^, the signalling pathways regulating these cellular processes are unclear. Furthermore, the specific molecules that prevent hydrocephalus by insuring proper ependymal cell formation remain to be discovered.

The present study has identified a novel hydrocephalus-causing gene, *Yap*, a downstream effector in the Hippo signalling pathway, as an important connection between an evolutionarily conserved signalling pathway and the pathogenesis of hydrocephalus. The Hippo pathway was first identified in *Drosophila* as a major regulator of tissue growth^10^. The components of this pathway are well conserved in mammals; most of the upstream regulators have been identified, but their regulation is more complex than in *Drosophila*^11^. Emerging data indicate that Yap has a critical role in tumorogenesis and tissue repair through interactions with other signalling pathways and responses to cellular cues, including those for cellular crowding and mechanical stresses^12^. A receptor-mediated signal from serum-derived lysophosphatidic acid (LPA) and related phospholipids has recently been shown to promote shuttling of Yap to the nucleus and subsequent transcriptional activation^13^,^14^. No previous study has demonstrated that the loss of cytoplasmic/junctional Yap due to LPA causes a deleterious outcome.

Our study demonstrates that nervous system-specific genetic ablation of *Yap* leads to early onset non-communicating hydrocephalus. Extensive phenotypic analysis establishes that Yap has a critical role in the generation of ependymal cells and the integrity of the apical lining of the aqueduct. Intriguingly, fetal haemorrhagic hydrocephalus induced by LPA, which mimics the Yap mutant phenotype, is accompanied by abnormal localization and reduction of Yap. Forced expression of phosphomimetic Yap (S112D), but not phospho-defective Yap (S112A), in LPA-treated animals partially restores N-Cadherin at the apical surface. Thus, our results demonstrate a novel function of cytoplasmic/junctional Yap in establishing and maintaining cellular and tissue integrity by supporting junction protein localization during normal development and after fetal brain injury.

## Results

### Loss of Yap in the nervous system causes hydrocephalus

**Figure 1:**
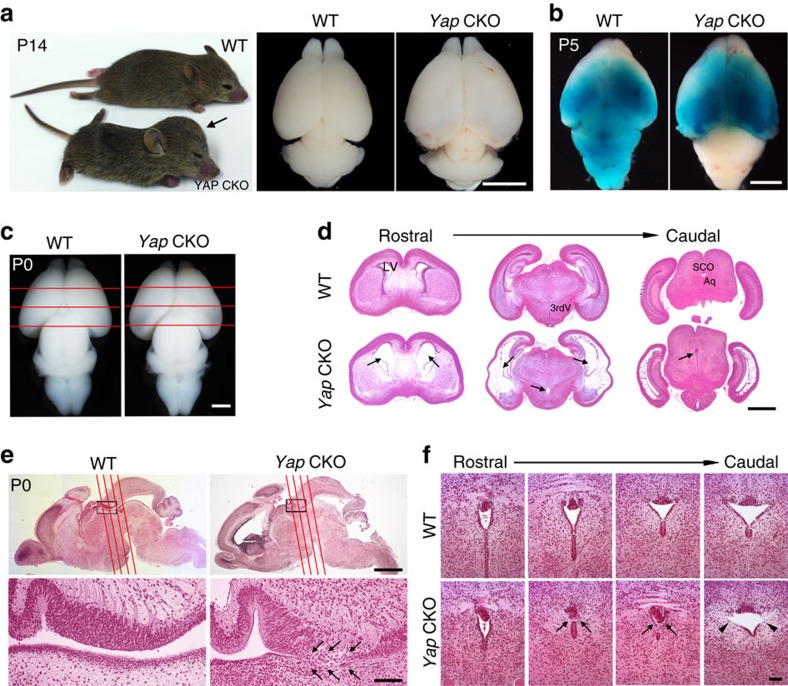
*Yap*-deficient mice develop non-communicating hydrocephalus. (**a**) At P14, all mutant mice display a dome-shaped head (arrow). The hemispheres of the *Yap* CKO mice are enlarged and the cerebellum are compressed compared with the WT control. (**b**) CSF flow is blocked at P5, shown by failure of dye injected into lateral ventricle to diffuse into fourth ventricle. (**c**) At P0, hydrocephalus phenotypes such as thinning of cortex and enlargement of caudal cerebral hemisphere are detectable in the *Yap* CKO. (**d**) Histological analysis of sections stained with H&E at P0 (rostral to caudal positions in **c**) demonstrates dilation of lateral ventricles and closure of aqueduct in the *Yap* CKO as compared with the WT (arrows). (**e**) Sagittal brain section at P0 reveals the obstructed area in the rostral aqueduct, which is enlarged in the lower panel. Four rostral to caudal planes (red lines) through the aqueduct with matching cross sections are shown in **f**. Scale bars, 5 mm (**a**); 3 mm (**b**); 1 mm (**c**,**d**); 2 mm (upper half of **e**), 100 μm (lower half of **e**); and 60 μm (**f**). 3rdV, third ventricle; Aq, aqueduct; LV, lateral ventricle; SCO, subcommissural organ.

Yap is highly expressed in the developing nervous system and acts as a downstream effector of NF2, regulating neural progenitor proliferation in the hippocampus^15^. However, the primary roles of Yap in nervous system development and the mechanism by which Yap acts in the pathogenesis of abnormal neural development remain elusive. To understand these issues, we generated a nervous system-specific *Yap* mutant using *Nestin Cre*. Remarkably, ablation of *Yap* caused a severe hydrocephalus phenotype (Fig. 1a) which began during late embryogenesis as a thinning of the caudal lateral cortex, was apparent at Postnatal (P) day 0 (Fig. 1c), and resulted in complete lethality around the age of weaning (Supplementary Fig. 1). To determine whether hydrocephalus in the *Yap* CKO (used throughout this manuscript to refer to *Yap*^*flox/flox*^; *Nestin Cre*) was non-communicating or communicating, we assessed whether dye injected into one lateral ventricle could diffuse freely into the fourth ventricle. In contrast to wild type (WT), dye failed to reach the fourth ventricle at P0, P3 (Supplementary Fig. 1) and P5 (Fig. 1b), providing direct evidence for physical blockage of the aqueduct and resulting non-communicating hydrocephalus. At P0, histological analysis at three rostral to caudal levels (Fig. 1c,d) revealed that the ventricles were most enlarged in the caudal section. Importantly, we found the rostral portion of the aqueduct to be closed, suggesting blockage of CSF flow in this area at P0 (Fig. 1d).

To further investigate the extent of the obstructed aqueduct area, we examined the mid-sagittal section, which bisects the aqueduct (Fig. 1e), and coronal sections in four different planes along the aqueduct (rostral to caudal, shown in red lines). Histological analysis of these sections enabled us to map the exact area of blockage in the *Yap* CKO (Fig. 1f). Interestingly, the ventral lining cells of the aqueduct were missing, and elongated dense cells comprising the dorsal subcommissural organ (SCO) were tilted in the blocked aqueduct area (Fig. 1f, low magnification picture can be found in Supplementary Fig. 1); these abnormalities are the likely cause of the CSF obstruction. Although the caudal portion of the aqueduct was widely open in both WT and *Yap* CKO at P0, lateral apical lining cells were largely absent in the *Yap* CKO (Fig. 1f), providing further evidence for defects of the ventricular lining due to missing ependymal cells. These results demonstrate that *Yap* deletion in the developing brain causes non-communicating hydrocephalus as early as P0 due to blockage of the rostral aqueduct.

### Yap is required for proper generation of ependymal cells

**Figure 2:**
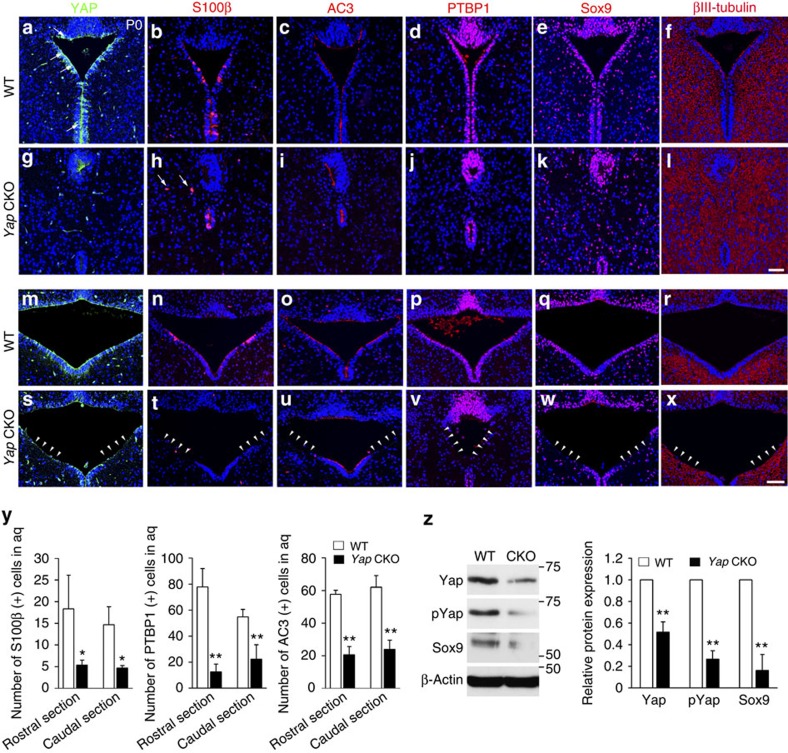
The apical integrity of ventral aqueduct is compromised in the absence of Yap. (**a**,**g**,**m**,**s**) At P0, ventral lining cells in the developing aqueduct, including ependymal cells and their precursors, express Yap (arrows). Mature ependymal cells expressing S100β (**b**,**h**,**n**,**t**,**y**), AC3 (**c**,**i**,**o**,**u**,**y**) or PTBP1 (**d**,**j**,**p**,**v**,**y**) are largely absent in the *Yap* CKO (arrow heads), whereas they are present in mature ependymal cells lining most of the ventricular surface of the aqueduct in WT. (**e**,**k**,**q**,**w**,**z**) Sox9 (+) progenitors/ependymal cell precursors are present but dispersed and reduced level of Sox9 protein is also detected likely due to the decreased number of Sox9 (+) cells. (**f**,**l**,**r**,**x**) βIII-tubulin (+) neuronal processes expand to the ventricular surface in the *Yap* CKO (arrow heads), whereas βIII-tubulin (+) processes are mainly excluded from the ventricular lining region in WT. For quantification of S100β (+), PTBP1 (+) and AC3 (+) cells, immuno-positive cells in the ventral lining of aqueduct (excluding SCO) were counted from the images (*n*=3). Two-tailed unpaired *t*-test reveals statistical significance (**P<0.01, *P<0.05). Error bars represent mean+s.e.m.; *n*=3. Scale bars, 50 μm.

To determine Yap's function in assuring aqueduct integrity, we first examined Yap expression in the rostral and caudal aqueduct because the aqueduct ventricular surface was obviously disrupted in the *Yap* CKO (Fig. 2a,g,m,s). At P0, Yap was highly expressed in ventricular lining cells, SCO (Fig. 2a) and choroid plexus (Supplementary Fig. 2), which are all involved in normal ventricular system function^16^,^17^. To test whether *Nestin Cre* is expressed in the choroid plexus or SCO at P0, Cre recombinase activity was monitored using a reporter line (Supplementary Fig. 2). A consistent finding was that neither SCO nor choroid plexus of *Yap* CKO demonstrated altered expression of Yap or obvious structural abnormalities, thus excluding their contribution to hydrocephalus formation. To eliminate the possibility that a mechanism independent of the aqueduct induces non-communicating hydrocephalus, we deleted *Yap* in other parts of the neuroepithelium by *Emx1 Cre* (expressed only in dorsal cortex) and *hGFAP Cre* (radial glia progenitors but not in the ventral midbrain). We observed neither non-communicating hydrocephalus nor aqueduct stenosis in either of these *Yap* CKO Cre lines (Supplementary Fig. 1), additional evidence that *Yap* deletion specifically from ependymal cells along the aqueduct causes these conditions.

To investigate the extent of ependymal cell loss in the absence of Yap, we examined cells expressing ependymal cell markers such as S100β and a cilia marker, adenylyl cyclase (AC) 3. Ependymal cells along the aqueduct mature earlier than elsewhere in the ventricular system, suggesting a critical role in maintaining aqueduct integrity and CSF flow^18^. Consistent with this idea, we observed that apical lining cells in the rostral aqueduct largely overlapped with S100β immunostaining at P0, while fewer cells expressed S100β in the caudal area (Fig. 2b,h,n,t). The enrichment of S100β (+) apical lining cells in the rostral aqueduct coincided closely with the region of aqueduct closure in the *Yap* CKO, which highlights the importance of early ependymal cell maturation for preventing aqueduct stenosis. In the *Yap* CKO, the number of S100β (+) cells was profoundly reduced, and some were displaced basally (Fig. 2h,t,y). Similarly, AC3-expressing cells were largely absent in the rostral and caudal aqueduct in the *Yap* CKO (Fig. 2i,u,y). Polypyrimidine tract-binding protein 1 (PTBP1)-labelled apical lining cells of the aqueduct were also significantly reduced in the *Yap* CKO compared with WT (Fig. 2d,j,p,v,y). We have found that the transcription factor, Sox9, is highly expressed in the apical lining cells in addition to other cells scattered in the midbrain. In the *Yap* CKO, Sox9-expressing cells in the apical lining were reduced, which was confirmed by reduced levels of Sox9 protein (Fig. 2e,k,q,w,z). Conversely, βIII-tubulin expressing neural processes fully occupied the aqueduct lining area in the absence of Yap, where they were largely excluded in the WT (Fig. 2f,l,r,x). Together, these observations support the notion that Yap is required for proper ependymal cell generation along the aqueduct, which is necessary to prevent aqueduct stenosis.

### Proliferation is impaired in the Yap deficient aqueduct

**Figure 3:**
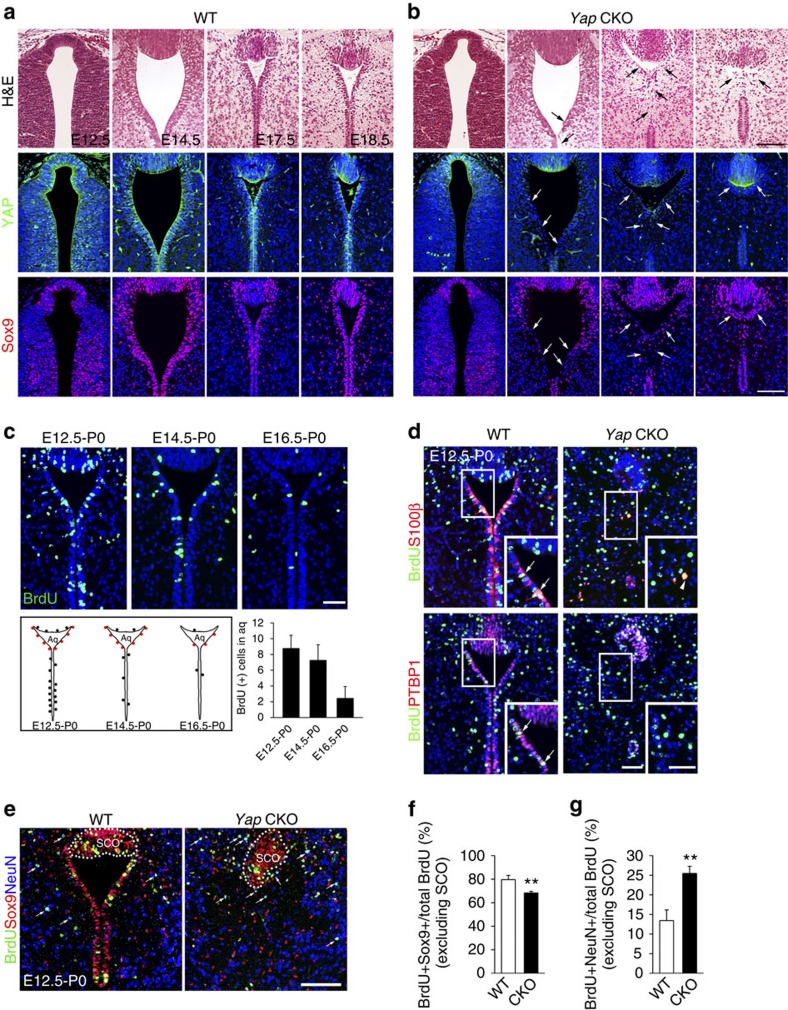
Yap is required for the generation of ependymal cells. (**a**,**b**) Histological analysis of a series of developmental stages reveals the progression of ependymal layer defects and subsequent aqueduct stenosis. Yap expression in the aqueduct of WT becomes restricted to ependymal cells during development, but in the *Yap* CKO, Yap expression remains only in the SCO, where *Nestin Cre* expression is absent. In WT, Sox9 is expressed in cells lining the ventricular wall and in cells scattered around aqueduct. In *Yap* CKO, Sox9 (+) cells are dispersed and found in the ventricular lining starting at E14.5 and later stages. (**c**) Birthdate of ependymal cells in the rostral aqueduct determined by analysis of BrdU (+) cells on P0 following BrdU injection on E12.5, E14.5 or E16.5. Schematic birthdate of apical lining cells (cells retaining high levels of BrdU) is shown as a dot; red dots indicate BrdU (+) cells in the ventricular lining of the aqueduct. Quantification of BrdU (+) cells demonstrates that most are born on E12.5 and E14.5. (**d**) At P0, the ependymal cell markers S100β and PTBP1 are co-expressed with BrdU administered on E12.5 in the rostral aqueduct of WT (arrows), but few cells are double positive for BrdU and S100β or PTBP1 in the *Yap* CKO (arrow head). (**e**–**g**) Fate analyses of BrdU retaining cells in WT and CKO revealed that the reduction of Sox9 (+) ependymal cells and glia progenitor cells but increased NeuN (+) neurons in the *Yap* CKO compared with WT. Quantification of BrdU (+) cells was done by counting postive cells in ventral lining of aqueduct excluding SCO (*n*=3). For quantification of BrdU (+) and Sox9 (+), or NeuN (+) cells, we counted single- or double-positive cells (excluding SCO) in the images (*n*=3). Two-tailed unpaired *t*-test reveals statistical significance (**P<0.01). Error bars represent s.e. of the mean.; *n*=3. Scale bars, 100 μm (**a**,**b**,**e**) and 50 μm (**c**,**d**).

To determine when cellular defects begin in the rostral aqueduct of *Yap* CKO, we undertook a comprehensive histological analysis at various developmental stages (Fig. 3a,b) and compared Yap expression in WT and *Yap* CKO (Fig. 3a,b and Supplementary Fig. 3). Progenitor cells expressed Yap in the aqueduct of WT, which was markedly reduced beginning at E12.5 in the *Yap* CKO aqueduct (Supplementary Fig. 3). Immunostaining for Yap (Fig. 3a,b) or phosphorylated Yap (S112) (Supplementary Fig. 3) showed that Yap expression in progenitors gradually became concentrated in the apical lining cells during development. *Yap* CKO first showed thinning and compromised integrity of the aqueduct apical lining at E14.5, whereas closure of the aqueduct, which would lead to the blockage of CSF flow, was observed as early as E18.5 in the narrow area of the rostral aqueduct (Fig. 3b). In the *Yap* CKO, expression of Sox9 and Nestin, which label cycling cells and possibly immature ependymal cells, also appeared to be reduced and dispersed (Fig. 3a,b and Supplementary Fig. 3).

To assess the contribution of reduced precursor production to ependymal cell loss, we studied the birthdates of ependymal cells along the aqueduct. For these experiments, we used reference points previously used to study ependymal cells in the subventricular zone, including E12.5, E14.5 and E16.5 (ref. 19). Most ependymal cells in the subventricular zone of the lateral ventricle are born during neurogenesis (E14.5 and E16.5), but maturation takes place postnatally. We injected bromodeoxyuridine (BrdU) at each time point in timed-pregnant females and analysed high [BrdU]-retaining cells in the rostral aqueduct at P0, when most of the apical lining cells become S100β (+) and PTBP (+). We found that most ependymal cells in the rostral aqueduct were born from E12.5 to E14.5 (Fig. 3c). Regardless of their birthdate, ventricular lining cells were not maintained, and few BrdU (+) cells expressing S100β were found in the *Yap* CKO at P0 (Fig. 3d). Most of the dividing BrdU (+) cells labelled at E12.5 expressed Sox9 and NeuN at P0 regardless of their position (Fig. 3e and Supplementary Fig. 4), including whether or not they were present in the ventricular lining; few showed overlapping staining for other cell markers, such as GFAP, Olig2, TH or Iba1 (Supplementary Fig. 4). Thus, reduced Sox9 and BrdU double-positive cells in the *Yap* CKO correlated with decreased numbers of BrdU (+) cells at P0 (Fig. 3e,f and Supplementary Fig. 4). Importantly, the fraction of BrdU (+) and NeuN (+) cells among BrdU (+) cells was increased by twofold in CKO (Fig. 3e,g), suggesting the overproduction of neurons at the expense of Sox9 (+) progenitors and ependymal cells.

**Figure 4:**
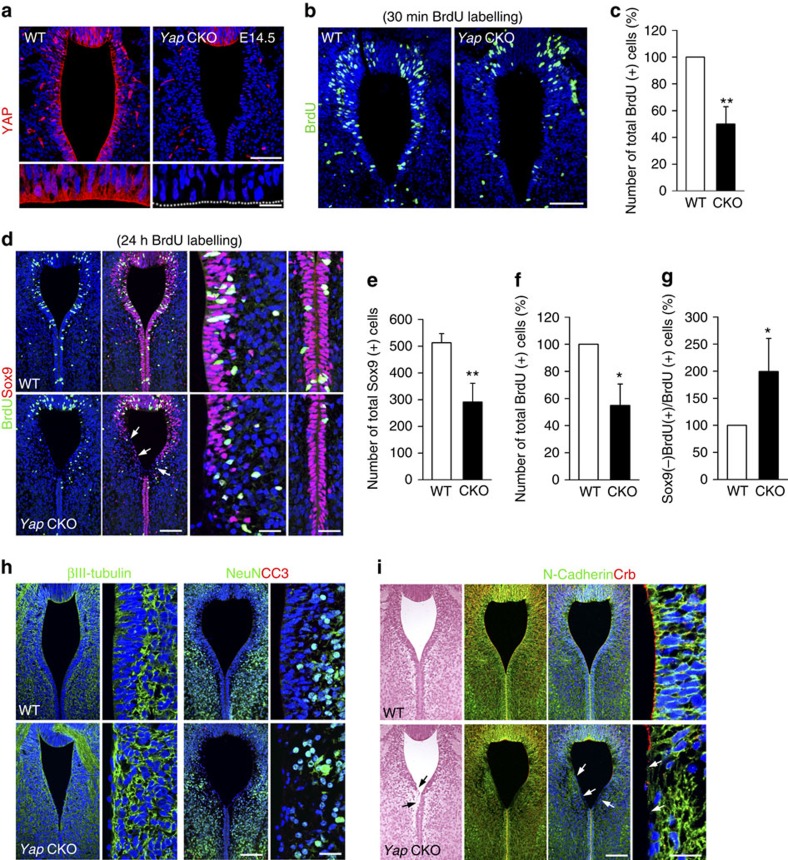
Yap is required for proliferation and polarity of ependymalprogenitor cells. (**a**) Yap is highly expressed in nucleus, cytoplasm and apical surface (enlarged picture) of progenitor cells, but is no longer detectable in the *Yap* CKO. (**b**,**c**) Compared with WT, *Yap* CKO shows fewer proliferating cells marked by BrdU incorporated by S-phase precursors at E14.5. (**d**–**g**) *Yap* CKO aqueduct contains fewer Sox9 (+) cells than WT at E14.5 (arrows). The fraction of cells exiting cell cycle (Sox9 (−) BrdU (+)/total BrdU (+) cells 24 h after BrdU labelling) is increased in the *Yap* CKO compared with WT. (**h**) βIII-tubulin (+) processes cover the ventricular surface of *Yap* CKO, whereas VZ is largely devoid of βIII-tubulin (+) processes in the WT. In the *Yap* CKO, NeuN (+) cells are also shifted to the VZ where progenitor cells are scarce. (**i**) adherens junction protein, N-Cadherin, which is concentrated in the apical endfeet in WT, are diminished in the *Yap* CKO (arrows). Apical polarity complex protein Crb, detected by pan-Crb protein antibody, has largely disappeared from the apical surface of the aqueduct in the *Yap* CKO (arrows). Quantification of BrdU (+) and Sox9 (+) cells was done by counting positive cells in the images, excluding SCO (*n*=3). Two-tailed unpaired *t*-test reveals statistical significance (**P<0.01 and *P<0.05). Error bars represent mean+s.e.m.; *n*=3. Scale bars, 50 and 20 μm (**a**); 50 μm (**b**); 100 and 20 μm (**d**,**h**,**i**).

To understand how Yap functions in progenitor cells to promote ependymal cell generation, we examined its expression pattern in the aqueduct at E14.5. In the WT, Yap was highly expressed in progenitors with both nuclear and cytoplasmic/junctional localization, which was strikingly diminished in the *Yap* CKO (Fig. 4a). To determine changes in the progenitor population, we first examined S-phase cells after BrdU pulse labelling. Yap loss significantly reduced the number of BrdU (+) S-phase cells compared with WT (Fig. 4b,c). We next determined the total number of Sox9 (+) cells in the ventral aqueduct and found that they too were significantly reduced in the *Yap* CKO compared with WT (Fig. 4d,e). To test whether the loss of proliferating cells was due to early cell cycle exit or cell death, we determined the fraction exiting the cell cycle 24 h after BrdU labelling. In the *Yap* CKO, the fraction of BrdU (+) cells that were no longer Sox9 (+) cells were twofold increased than that of WT (Fig. 4d,g), evidence that *Yap*-deficient progenitors exited the cell cycle prematurely. Importantly, βIII-tubulin (+) neural processes occupied the ventricular area and apical surface in the *Yap* CKO, indicating premature neuronal differentiation (Fig. 4h). Consistent with this pattern of βIII-tubulin expression, NeuN (+) neurons were found in the ventricular area of *Yap* CKO, where NeuN (−) proliferating cells were located in WT (Fig. 4h). Staining for cleaved caspase 3 expression to monitor cell death demonstrated no obvious cell death in either WT or CKO (Fig. 4h and Supplementary Fig. 5). Together, these results indicate that Yap is required for aqueduct apical lining integrity and that it functions by preventing ependymal cell progenitors from exiting the cell cycle.

### Yap is required for apical attachment and cell shape

**Figure 5:**
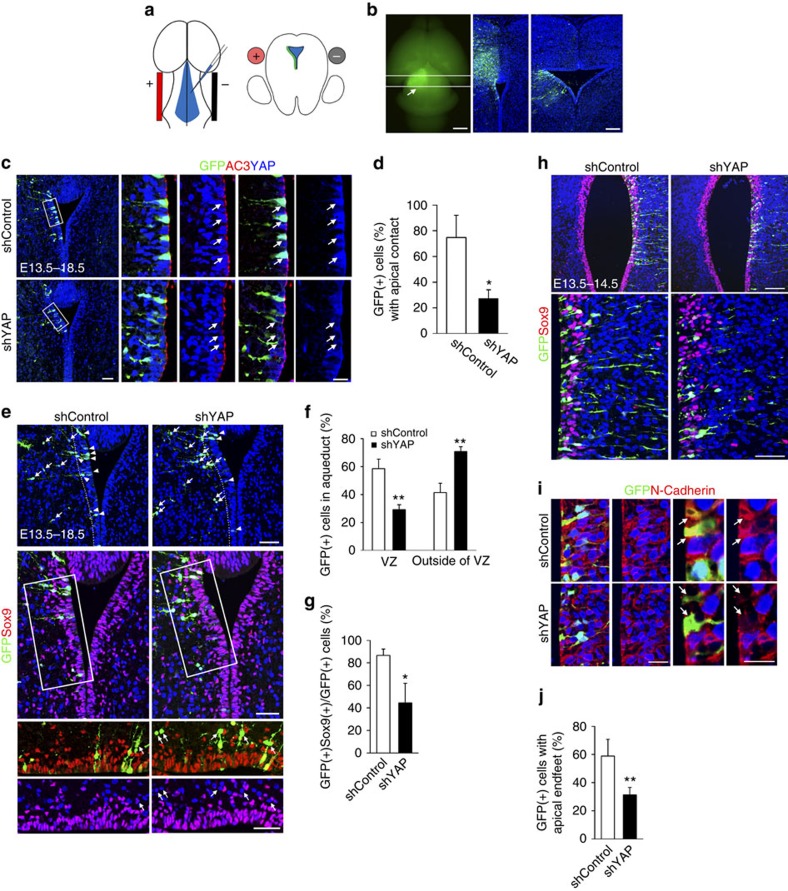
Yap is necessary to maintain apical attachment of progenitor cells. (**a**) DNA constructs are electroporated to the developing aqueduct by injecting DNA and applying an electrical pulse across the aqueduct. (**b**) Cross-section view depicts the distribution of GFP (+) electroporated cells in the rostral and caudal regions. (**c**,**d**) Most *Yap* KD cells lose their apical contact and are no longer maintained in the VZ after 5 days (E13.5–E18.5). Apical lining cells demarcated by the cilia marker AC3 in the electroporated area show prominent loss of Yap (blue) as compared with those in the control shRNA electroporated area. (**e**–**g**) >60% of *Yap* KD cells, but only 40% of control shRNA-expressing cells, are located outside of VZ (arrows) (*n*=3). Most *Yap* KD cells, but only 10% of control shRNA electroporated cells, are Sox9 (−) cells (arrows) (*n*=3). (**h**) Most electroporated cells express Sox9 and remain in the VZ 24 h after electroporation at E13.5, but they do not maintain apical endfeet. (**i**,**j**) The remaining apical endfeet of *Yap* KD show only a few spots of N-Cadherin labelling, whereas apical endfeet are densely labelled for N-Cadherin in control shRNA electroporated cells (arrows). Two-tailed unpaired *t*-test reveals statistical significance (**P<0.01 and *P<0.05). Scale bars, 1 mm and 50 μm (**b**); 50 and 20 μm (**c**); 50 μm (**e**); 100 and 50 μm (**h**); 100 and 20 μm (**i**).

Because Sox9 (+) cells were widely dispersed and the apical surface of the ventricular lining of the aqueduct was disrupted, we used junction protein localization to assay tissue integrity. At E14.5, *Yap* CKO showed strikingly reduced junctional concentrations of N-Cadherin at the apical endfeet, which were clearly visible in the apical portion of the cell–cell contact region in the WT (Fig. 4i). More obviously, the distribution of Crb polarity complex protein, which has a distinctive concentration at the apical surface in the WT, was markedly reduced in the *Yap* CKO (Fig. 4i). Because junctional disruption was profound in the *Yap* CKO and junction protein defects can cause proliferation defects, we reasoned that progenitor depletion may be secondary to tissue integrity defects caused by Yap loss. To investigate the primary function of Yap in tissue integrity and progenitor loss, we used *Yap* knockdown (KD) in the developing aqueduct to investigate cell-autonomous defects. To target ependymal cell precursors, we administered *Yap* shRNA, which can effectively reduce Yap in cell culture (Supplementary Fig. 6), to the developing aqueduct at E13.5. DNA constructs were injected into the aqueductal region, and an electrical pulse was applied across the midbrain (Fig. 5a,b). To visualize cellular features such as apical endfeet and shape of the targeted cells, we co-electroporated green fluorescent protein (GFP)-expressing vectors with *Yap* shRNA or with control shRNA. At E18.5, after 5 days *in utero*, normal ependymal progenitor cells had a wider apical surface than in KD, part of which overlapped with cilia (Fig. 5c). A significant fraction of *Yap* KD cells lost their apical contact and most were not retained in the ventricular zone (VZ). In contrast, many control shRNA-expressing cells remained in the VZ (Fig. 5c,d). Interestingly, *Yap* KD cells located outside VZ, but only rare control shRNA-expressing cells, often did not express Sox9, suggesting that transcriptional regulation or cell fate may change when Yap is reduced (Fig. 5e–g). Remarkably, the loss of apical attachment preceded loss of Sox9 expression as apical shrinkage of *Yap* KD-expressing cells were detected 24 h after electroporation while Sox9 was unaltered (Fig. 5h–j). Furthermore, even in *Yap* KD cells with seemingly normal apical endfeet, the reduction of N-Cadherin was apparent, suggesting that losing junctional complex will eventually lead to the detachment of endfeet from apical surface (Fig. 5i). These results support the notion that the disruption of apical junction and apical attachment is one of the most immediate cellular changes after Yap reduction. Taken together, the establishment/maintenance of polarized cell shape and adhesion of ependymal cell progenitors require intact Yap function and the lack of normal Yap function may critically contribute to aqueduct stenosis by compromising the generation of the ependymal cell layer.

### Yap maintains cellular architecture of cortical progenitors

**Figure 6:**
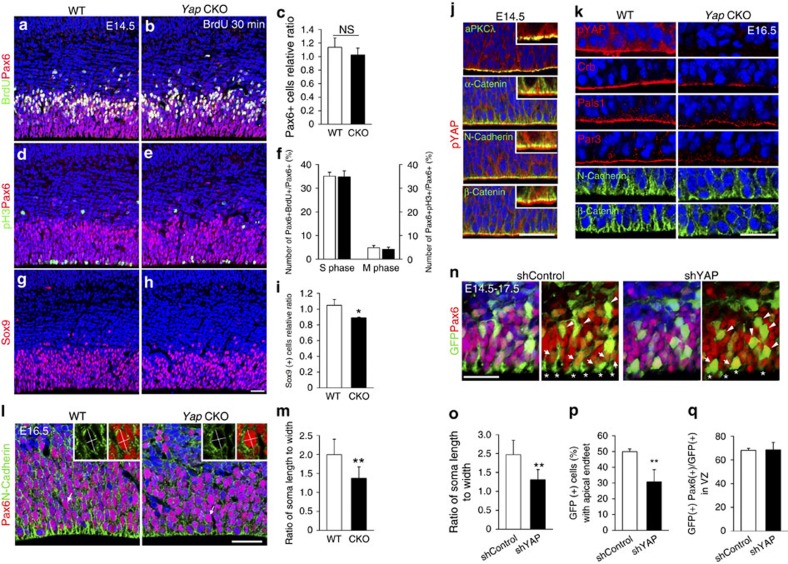
Yap loss causes defects in cell shape and apical attachment of cortical progenitors. (**a**–**f**) At E14.5, the *Yap* CKO contains slightly fewer Pax6 (+) cells than WT, although the fractions of BrdU (+) S-phase cells and of pH3 (+) M-phase cells among total Pax6 cells are not significantly different. (**g**–**i**) Sox9 (+) cells are reduced in number in the absence of Yap. (**j**) In the cortical epithelium, pYap is localized in the cytoplasm and highly enriched at the apical junction; this distribution overlaps with aPKCλ and is apical to adherens junction marked by N-Cadherin, β-Catenin or α-Catenin. (**k**) N-Cadherin or β-Catenin labelling in apical endfeet is reduced and less concentrated in the *Yap* CKO than in the WT. Apical polarity complex proteins, Crb, Pals1 and Par3 are reduced at the apical surface when pYap is largely depleted at E16.5. (**l**,**m**) At E16.5, in the *Yap* CKO, the ratio of soma length to width of Pax6 (+) apical progenitor cells is reduced as compared with WT. (**n**–**q**) Fewer *Yap* KD cells have apical endfeet contacting the apical surface than control shRNA-expressing cells (arrows and *). *Yap* KD cells have a rounded cell shape (cellular length to width ratio is close to 1, arrow head). However, there is no difference in the proportion of Pax6 (+) cells among total GFP-expressing cells in the VZ. Two-tailed unpaired *t*-test reveals statistical significance (**P<0.01 and *P<0.05). Scale bars, 50 μm (**a**,**b**,**d**,**e**,**g**–**j**); 30 μm (**n**).

To determine whether Yap's essential function in tissue integrity and cellular proliferation is conserved in other CNS tissues, we extended our study to the developing cortex. First, to detect proliferation defects of cortical progenitors, we undertook a comprehensive analysis at E14.5, when Yap and pYAP are largely missing in the CKO (Supplementary Fig. 7). The number of cortical progenitor cells labelled by Pax6 was slightly reduced compared with WT (Fig. 6a–c). However, we could not find any significant difference in the fraction of S-phase cells marked by BrdU pulse labelling or in M-phase cells labelled by pH3 among total Pax6 (+) progenitor cells (Fig. 6a–f). Since Sox9 (+) cells were severely decreased in the developing aqueduct, we also examined Sox9-expressing cells in cortex. Sox9 (+) cells were in the VZ but fewer than Pax6 (+) cells, suggesting that Sox9 represents a subpopulation of cortical progenitors. Importantly, Sox9 (+) cells were reduced in number in the CKO as compared with WT (Fig. 6g–i), indicating that the size of the progenitor subpopulation changes in the absence of Yap. However, the neuronal production was not significantly altered in the *Yap* CKO at P0 despite cortical thinning likely due to pressure from accumulated CSF (Supplementary Fig. 8). Interestingly, this pressure mainly reduced the thickness of the intermediate zone, not of the cortical plate. Furthermore, the progenitor pools labelled by BLBP and NICD were also reduced in thickness at P0, and also contributed to the thinning of cortex (Supplementary Fig. 8). Thus, although Yap loss did not distinctively affect cortical neurogenesis, the progenitor population was reduced at later stages.

To examine cellular phenotype and tissue integrity, we next analysed Yap expression in detail. Consistent with previously reported expression patterns^15^, Yap and pYap (S112, human S127) were highly expressed in neural progenitors (Supplementary Figs 7 and 8). As in the aqueduct, pYap was enriched in the apical surface of neuroepithelium and extensively overlapped with adherens junction proteins such as β-Catenin and N-Cadherin and with the tight junction protein aPKCλ (Fig. 6j). Similar to aqueduct, the localization pattern of adherens junction proteins was disorganized and/or reduced in the *Yap* CKO at E14.5 and E16.5 (Fig. 6m and Supplementary Fig. 7). Furthermore, apical complex proteins Crb, Pals1, Par3 and aPKCλ were substantially reduced at the *Yap* CKO apical surface (Fig. 6k and Supplementary Fig. 7). However, the disruption of junction protein localization was less severe in cortex than in aqueduct, consistent with the lack of profound phenotype of progenitor loss and neurogenesis defects.

At E16.5, *Yap* mutant cells began to be rounder and less elongated along the apical-basal axis than WT (Fig. 6l). To determine the extent of the cell shape changes, we analysed the soma length to width ratio of Pax6 (+) apical progenitors outlined with N-Cadherin. The ratio between WT (2.3) and CKO (1.5) was significantly different, evidence for cell shape changes in the absence of Yap (Fig. 6l,m). To further investigate whether Yap is required for establishing and/or maintaining localization of apical junction proteins and cellular architecture in a cell-autonomous fashion, we used *Yap* shRNA to create a mosaic cortex in which Yap activity was partially inactivated in a subset of progenitor cells. Cortical cells electroporated with *Yap* shRNA plasmid at E14.5 were marked by GFP and collected after 3 days *in utero*. *Yap* KD cells showed apical endfeet that were abnormally shortened or detached from the ventral surface (Fig. 6n,p). Moreover, most *Yap* KD progenitors were round and failed to achieve the normal elongated shape (Fig. 6n,o). However, the number of Pax6 (+) *Yap* KD cells was not altered compared with control cells, which is similar to previous results in the *Yap* CKO cortex (Fig. 6n,q). Collectively, these results show a less prominent role for Yap in the proliferation of cortical progenitors than was suggested by previous studies, in which overexpression of nuclear Yap in the developing cortex profoundly stimulated progenitor proliferation^20^. Importantly, our genetic study shows that Yap has a critical role in maintaining cellular architecture, polarity and integrity of the cortical epithelium by maintaining adherens junction and apical complex proteins at the junction.

### Loss of Yap contributes to LPA-induced hydrocephalus

**Figure 7:**
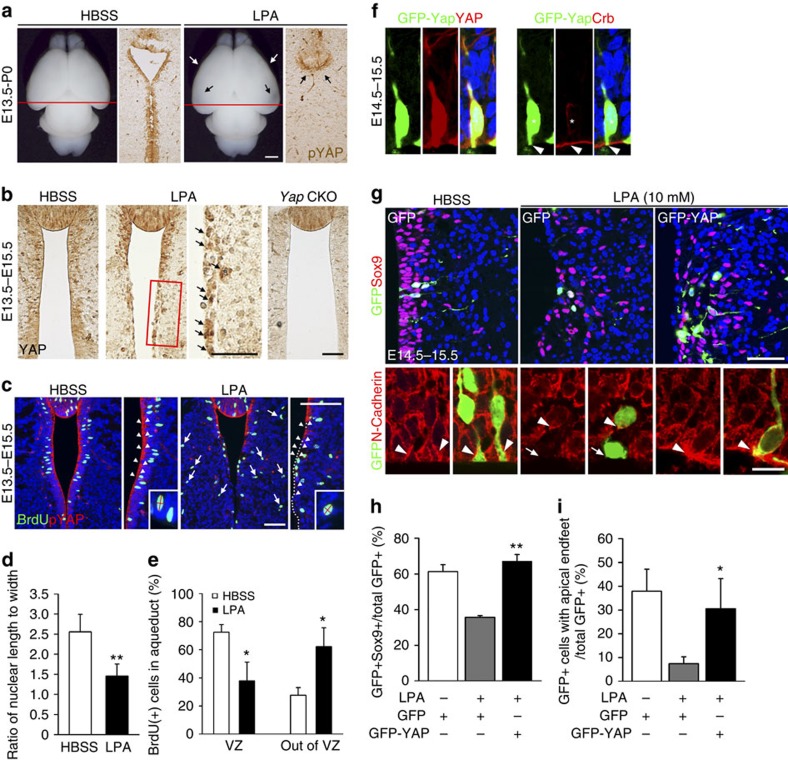
Forced Yap expression reverses LPA-induced cellular defects. (**a**) LPA injection into the lateral ventricle at E13.5 produces changes analogous to those of the *Yap* CKO at P0: decreased Yap expression in ependymal cells along the aqueduct. (**b**) At E15.5, Yap is already decreased in the cytoplasm/junction of ependymal cell precursors, but nuclear Yap is present in a subset of apical lining cells (arrows) in the LPA-treated embryos. (**c**) pYap proteins are undetectable in the ventral aqueduct in the *Yap* CKO at E15.5 (arrow head). (**d**) Following LPA administration at E13.5, nuclear shape becomes rounder (length to width ratio close to 1 (1.3), than in control HBSS-treated animals (2.5). (**e**) Many BrdU (+) S-phase cells are scattered outside of VZ in mice receiving LPA, compared with control. (**f**) On electroporation, GFP-Yap fusion protein recapitulates Yap localization in nucleus, junction and cytoplasm. (**g**–**i**) GFP-Yap electroporated into the aqueduct before LPA injection into a lateral ventricle restore Sox9 expression and apical attachment to levels comparable to those of HBSS-treated control cells. Quantification of BrdU (+), GFP (+) or Sox9 (+) and GFP (+) cells was done by counting single- or double-positive cells in the images (*n*=3). Two-tailed unpaired *t*-test reveals statistical significance (**P<0.01 and *P<0.05). Error bar represent mean+s.e.m.; *n*=3. Scale bars, 1 mm (**a**); 50 μm (**b**,**c**); 50 and 10 μm (**g**).

To explore whether deregulated Yap is involved in injury-mediated fetal hydrocephalus, we tested Yap's role in LPA-induced hydrocephaly. Because LPA is a blood-borne bioactive lipid found in plasma/serum, LPA-mediated hydrocephalus models fetal haemorrhagic hydrocephalus^21^. Recent studies identified LPA as an extracellular upstream regulator of Yap that reduces Yap phosphorylation and increases transcriptional activation via G12/13-coupled receptors^14^. Importantly, LPA injection into developing cortices causes hydrocephalus, which can be partially abolished by genetic ablation of LPA receptors *Lpar1/2* (ref. 21). Hydrocephalus induced by injecting LPA into developing cortices at E13.5, is strikingly similar to the *Yap* CKO phenotype. These similarities, which include complete closure of the rostral aqueduct at P0 (Fig. 7a and Supplementary Fig. 10) and ependymal cell loss, raise the potential link between LPA and Yap. To explore whether Yap is downstream effector of LPA-mediated hydrocephalus, we examined changes of Yap localization in aqueduct at P0 when hydrocephalus is apparent after injecting LPA at E13.5. We discovered that pYap expression was markedly reduced at P0 (Fig. 7a). Intriguingly, nuclear Yap remained in a subset of apical lining cells at E15.5, two days after LPA injection and before rostral aqueduct closure, compared with near-complete absence in the *Yap* CKO (Fig. 7b,c). Next, we explored whether LPA treatment causes similar cellular phenotype to that of *Yap*-deficient cells in the developing aqueduct. We examined the shape of BrdU (+) S-phase cells after pulse labelling and found that cells became rounder, with a length to width ratio close to 1.3; the ratio of WT nuclei was ∼2.5 (Fig. 7c,d). LPA treatment also led to ectopic proliferating cells outside of VZ, shown by a significantly increased number of displaced BrdU(+) cells in the aqueduct (Fig. 7c,e). Collectively, LPA-induced cellular phenotype is similar to that of *Yap* mutants.

To determine the contribution of Yap loss as a downstream effect of LPA-induced injury, we carried out rescue experiments. For these experiments, we used a GFP-Yap fusion protein that localizes to the nucleus, cytoplasm and junction, representing the full spectrum of known Yap subcellular distribution (Fig. 7f). We electroporated GFP-Yap fusion protein into the developing aqueduct before LPA injection into the lateral cortex on E13.5. To evaluate the extent of rescue effects of LPA, we measured apical attachment of apical endfeet and Sox9 expression as loss of Sox9-expressing cells and reduced concentrations of junction proteins at the apical endfeet were additional consistent observations after LPA administration (Fig. 7g–i). Remarkably, 2 days after electroporation, 60% of GFP (+) *Yap*-overexpressing cells in the LPA-treated embryos showed Sox9 expression, which is similar to that of control HBSS-treated animals (Fig. 7g,h). Furthermore, N-Cadherin enriched apical endfeet in *Yap*-overexpressing cells remained relatively intact comparable to those of HBSS-treated animals, although tissue polarity was severely compromised by LPA administration (Fig. 7g,i). Collectively, our data suggest that LPA-induced fetal hydrocephaly is mediated at least in part through Yap loss, which impairs cellular junctional integrity and reduces progenitor cells in the developing aqueduct.

### Nuclear function of Yap is not required for junctional integrity

**Figure 8:**
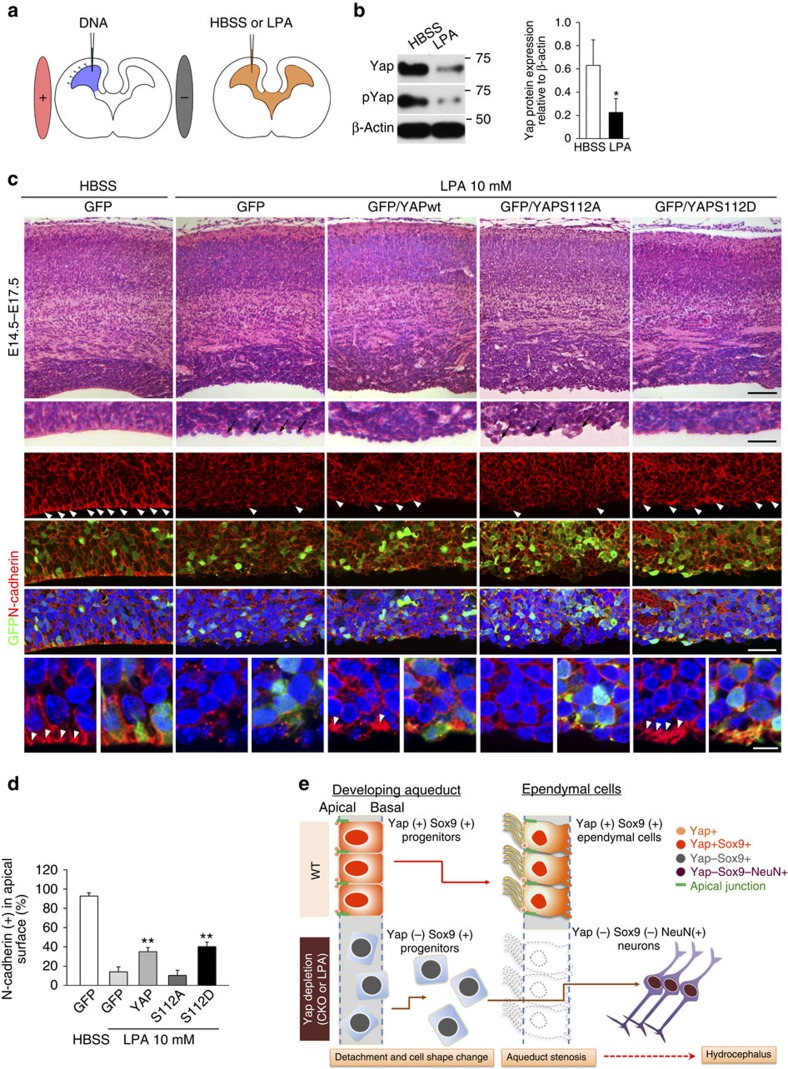
LPA-induced junction defects are partly rescued by forced expression of Yap. (**a**) Schematic of the rescue experiments: electroporation of *Yap* or *Yap* mutant constructs to the developing cortices followed by LPA administration to a lateral ventricle. (**b**) Yap and pYap are significantly reduced in the cortex 1 day after LPA injection into lateral ventricle at E13.5. β-actin was used as a loading control. (**c**) Histological analysis of an electroporated area of cortex after LPA reveals disruption of the ventricular surface: the apical lining of cells is segregated and lacks lateral attachment to adjacent cells. Number of protruding cells in the apical surface is reduced by *Yap* overexpression and more prominently by phosphomimetic *Yap* (S112D) expression. Phospho-defective *Yap* (S112A) expression fails to reduce these protruding apical surface cells, but expansion of VZ is evident. N-Cadherin localization at the cell junction and apical surface (arrow heads) is markedly decreased by LPA as compared with control (HBSS treated), and is partially restored by forced expression of *Yap* and phosphomimetic *Yap* (S112D), but not by phospho-defective *Yap* (S112A). (**d**) Number of N-Cadherin (+) cells at the apical surface counted and compared. (**e**) Schematic showing that loss of Yap by genetic deletion or LPA treatment results in disrupted junction proteins and a failure to form the ependymal layer. Two-tailed unpaired *t*-test reveals statistical significance (**P<0.01 and *P<0.05). Error bars represent mean+s.e.m.; *n*=3. Scale bars, 100, 40 and 10 μm (**c**).

Because LPA promotes nuclear translocation of Yap, we reasoned that reduced cytoplasmic/junction Yap rather than an impaired nuclear function of Yap caused the junctional defect phenotypes in LPA-treated tissues. This would imply a novel function for Yap in establishing and/or maintaining cellular polarity and adhesion. To test this possibility, we generated phosphomimetic *Yap* mutant (S112D), which partially mimics pYap, and phospho-defective *Yap* mutant (S112A) under the control of CAG promoter (Supplementary Fig. 9). As expected, our luciferase reporter assay showed transcriptional activation to be more efficient with the phospho-defective form of Yap than in WT or with the phosphomimetic form (Supplementary Fig. 9). If the LPA-mediated cellular phenotype is caused by loss of non-nuclear function of Yap, phosphomimetic Yap expression should rescue, at least partially, the junction phenotypes induced by LPA. For this rescue experiment, we used cortical epithelium since many cells can be targeted and the LPA response is robust and rapid. We observed induced cellular phenotypes in the cortices 2 and 4 days after LPA injection. The phenotypes included disruption of cell and tissue polarity, demonstrated by lack of Crb at the apical surface and rosette formation (Supplementary Fig. 10). More importantly, LPA treatment rapidly reduced Yap by as early as 24 h in the aqueduct (Fig. 8a,b). Furthermore, LPA-mediated cellular changes were well characterized in previous studies. For instance, loss of N-Cadherin at the junction and apical surface is one of the prominent and immediate effects of exposure to LPA in the CSF^21^ (Supplementary Figs 11 and 12). We therefore examined the restoration of N-Cadherin following electroporation of Yap constructs. We found that, when *Yap* or phosphomimetic *Yap* was electroporated into a lateral ventricle at E14.5, localization of N-Cadherin at the apical surface was partially restored (Fig. 8c,d). Furthermore, histological analysis revealed that the apical surface was relatively intact and that cells were less segregated than those of LPA-treated embryos electroporated with vector control or phospho-defective *Yap* (Fig. 8c). Importantly, phosphomimetic *Yap* rescued N-Cadherin localization at the apical surface more effectively than WT *Yap*. Conversely, forced expression of phospho-defective *Yap* did not rescue cellular integrity and failed to retain N-Cadherin at the junction (Fig. 8c,d). These results suggest the potential role of cytoplasmic/junction Yap loss in the pathology induced by LPA. Of note, *Yap* overexpression did not restore apical complex protein Crb in the apical surface, and Pax6 (+) apical progenitors and Tbr2 (+) basal progenitors remained scattered despite *Yap* or phosphomimetic *Yap* overexpression (Supplementary Fig. 11). These observations suggest that forced Yap expression is most efficient at recruiting N-Cadherin to the apical surface. Accordingly, *Yap* overexpression increases pYap at the apical surface and cell–cell junctions in LPA-treated animals, and N-Cadherin restoration coincides with concentrated pYap (Supplementary Fig. 12). Together, these findings suggest that LPA-induced disruption of cellular integrity is mediated, at least in part, by deregulated Yap expression and loss of Yap at the apical junction but not by enhanced transcriptional activation.

## Discussion

Our investigation of the pathophysiological cause and course of hydrocephalus generated by deleting the new hydrocephalus gene, *Yap*, has identified its critical function in apical junction integrity, generating ependymal cells via coordinated production from progenitors and maintaining cellular shape along the aqueduct. We also established that the mechanism by which Yap couples injury signals in the CSF to cellular responses entails *Yap* depletion and subsequent loss of cellular polarity and adhesion. Consequently, *Yap*-deficient progenitor cells in the aqueduct detach from the ventricular lining, lose expression of Sox9 and mainly become neurons, resulting in failure to form ependymal layer (Fig. 8e). These results highlight a new role of Yap as a key mediator of an injury signalling cascade that leads to non-communicating hydrocephalus. Thus, our study may provide the basis for the development of a drug for injury-mediated hydrocephalus that is specifically targeted to enhance Yap function and distribution.

Our study demonstrated that the multifaceted transcription activator, Yap, has an essential role in the development of ependymal cells along the aqueduct. It may integrate extrinsic signalling and cell contact information to establish the integrity of the ventricular system. The nuclear function of Yap has been extensively studied and lists of its target genes and interacting transcription partners have grown significantly in recent years^11^,^22^,^23^,^24^,^25^. Nuclear Yap controls proliferation and stem cell self-renewal in various tissues^12^, supporting its importance for progenitor proliferation. Accordingly, we detected nuclear Yap in both progenitor cells and a subpopulation of mature ependymal cells along the aqueduct (Fig. 2). Because Sox9 expression can be regulated by Yap-TEAD transcriptional factor as shown in other tissues such as liver^26^ and because we found Sox9-expressing cells to be reduced in both developing cortex and aqueduct, the loss of nuclear function may have contributed to the reduction of the Sox9 (+) subpopulation of progenitors. However, analyses of cortical progenitor cells with *Yap* loss-of-function did not show profound defects in proliferation despite a report that nuclear Yap can elicit marked progenitor pool expansion in skin^27^. Furthermore, although Yap function in cell survival via transcriptional activation of genes like *survivin* is also well documented in many systems^28^, our finding demonstrated that Yap has a less prominent role in cortical neurogenesis and survival. It is possible that the timing or efficiency of *Nestin Cre* or shRNA-mediated elimination of Yap may not be sufficiently robust to reveal its critical function in regulating cortical progenitor proliferation; we often detected residual nuclear Yap in the *Yap* CKO even at E16.5, when pYap was largely absent. Alternatively, the closely related homolog, Taz, may have a redundant role in cortical progenitor proliferation although loss of Yap is sufficient for causing hydrocephaly.

Our study supports the critical requirement for Yap in the cytoplasm/junction to maintain junctional complexes. Yap localization in the cytoplasm and at apical junction in neuroepithelium may also allow multiple interactions between signalling components as the apical junction serves as a critical site for signal reception and integration and contains many signalling components, including Notch receptors and Hippo-Yap signalling proteins. Furthermore, previous studies provide evidence of Yap function in the cytoplasm to sequester SMAD complexes^29^ or inhibit Wnt signalling^30^. As Wnt and Notch signalling have important roles in the decision of neural progenitor proliferation and differentiation^31^, cytoplasmic/junctional Yap may be involved in modulating these signalling pathways. Future studies investigating a potential interaction between these signalling molecules and Yap in cytoplasm/junction may yield additional insights into Yap function in neural/ependymal cell development.

Our phenotypic analyses showed that Yap mutant-induced hydrocephalus resembles the phenotype of the severe early onset congenital hydrocephalus in humans. More than 10 loci are now associated with congenital hydrocephalus in humans, including the X-linked hydrocephalus gene that encodes the cell adhesion molecule, *L1-CAM* (ref. 32). Although these identified genetic causes attest to the critical significance of normal cell adhesion, polarity and cytoskeletal organization in preventing hydrocephalus, the cell type and the extent of the defects responsible for hydrocephalus have not been established. The present study identifies the primary cause of a subtype of non-communicating hydrocephalus as defects in ependymal cells along the aqueduct. Furthermore, our observation that *Yap* KD ependymal cells/precursors are detached from the apical surface demonstrates the critical importance of adhesion for apical surface preservation. Although Yap localization and function have been described at cell junctions in epithelial tissues, no previous report has claimed that it has a role in junctional integrity^33^. However, our recent study of the lens epithelium^34^ and the current investigation suggest that Yap (especially pYap) influences cellular adhesion within the epithelium, cell shape and polarity by regulating the localization of junction proteins. Our results demonstrating an overlapping distribution of pYap and junction proteins and the restoration of N-Cadherin by forced Yap expression suggest that Yap (preferably pYAP at S112) actively recruits and retains junction proteins in the neuroepithelium. This function of supporting adhesive interactions between ependymal cells may be critical in preventing hydrocephalus. This interpretation is further supported by the report that deletion of *Dlg5* causes hydrocephalus due to impaired trafficking of the Catenin–Cadherin complex to the cell surface^9^. Thus, our observations may provide evidence for a novel scaffolding function of Yap assembling or maintaining junction proteins necessary for cell adhesion and reveal the critical molecular basis for the ependymal cell integrity that precludes non-communicating hydrocephalus.

Hydrocephalus is also a consistent feature of mice carrying mutation in polarity complex genes including *Lgl1*, *Cdc42* and *aPKCλ*, which also act as upstream regulators of Hippo-Yap signalling^35^,^36^,^37^,^38^. Although homologues of *Lgl1* and *aPKC* in *Drosophila* regulate Hippo (*hpo*) kinase activity by modulating dRassf inhibition of *hpo*^39^, we found that double conditional knockout of *Mst1/2*, the mammalian homolog of *hpo*, with *Nestin Cre* did not elicit a hydrocephalus phenotype (unpublished data). Thus, these upstream regulators may not work through Hippo kinase, as in *Drosophila*, but through a pathway that regulates cell polarity and adhesion. This idea receives support from the observation that apical polarity complex, including aPKC and basal complex protein Lgl1 have opposing roles in controlling proliferation of cortical progenitors but that both affect tissue polarity and integrity^40^. Furthermore, the hydrocephaly phenotype of *Cdc42* mutant is very similar to that of *Yap* mutant^41^, and the cortex specific mutants of *Cdc42* show massive detachment of apical progenitor cells, which become basal progenitor cells^42^. Similarly, previous work with *hyh* (hydrocephalus with hop gait) mice showed that ependymal cells were denuded and that apical junction proteins were substantially reduced at the apical surface of cortical neuroepithelium^43^. The notion that impaired tissue integrity and adhesion are common to all of these mutants despite other diverse features that depend on cellular contexts supports their identification as primary cellular defects responsible for hydrocephaly.

Our study establishes that Yap mediates injury signals by altering its subcellular localization and/or stability. LPA appears in the CSF immediately after traumatic brain^44^ or spinal cord injury^45^, and has a critical role in initiating pathogenic signalling pathways. The hydrocephalus phenotype of *Myo9a* KO is remarkably similar to that mediated by LPA and *Yap* CKO; it also shows closure of the caudal third ventricle and rostral aqueduct^46^. Myo9a has a GAP domain that converts Rho-GTP to GDP and thereby inhibits ROCK, a downstream component of the LPA/G protein-coupled receptor-mediated signalling cascade. ROCK inhibitors such as Y-27632 can therefore rescue hydrocephalus induced by LPA and the *Myo9a* KO^21^,^46^. Since LPA-mediated injury can elicit changes in gene expression^47^,^48^, it is possible that abnormal gene expression mediated by nuclear Yap could be responsible for the pathogenesis of hydrocephalus. However, on LPA treatment, loss of Sox9, immediate cell shape change and significant loss of total Yap protein (although nuclear Yap remains in a subpopulation) suggest that nuclear Yap may not have a prominent role in LPA-induced cellular changes. Instead, it is possible that decreased nuclear Yap may be responsible for the loss of Sox9-expressing cells as forced expression of phospho-defective Yap increases the VZ thickness (Fig. 8c). Importantly, the loss of pYap and apical surface integrity corresponds with N-Cadherin loss, one of the most prominent and immediate cellular changes following LPA treatment. Despite efficient transcription activation, phospho-defective Yap mutants cannot rescue the junctional defects, supporting the idea that loss of junctional and cytoplasmic pYap/Yap makes a major contribution to LPA-induced impairment of ependymal cell integrity. In accordance with the notion that pYap may exert a crucial function, a recent report showed that phosphomimetic Yap (S127D) can limit the activity of Wnt reporter^49^. Thus, phosphorylated Yap in the cytoplasm/junction may have a more important signalling role in various cellular contexts than has been previously appreciated. Importantly, we provide evidence that the loss of junctional/cytoplasmic Yap in ependymal cells may have a prominent role in pathologic outcomes. It is therefore possible that overexpression of phosphomimetic *Yap*, Lats 1/2 kinase or another kinase responsible for phosphorylation of Yap, or drugs that promote Yap phosphorylation or junctional retention can be similarly effective in preventing or reversing certain forms of congenital and traumatic non-communicating hydrocephalus.

## Methods

### Mice

All animal experiments were performed in accordance with the guidelines of the Institutional Animal Care and Use Committee of Temple University School of Medicine. The *Yap* floxed allele was obtained from Dr Olson (Southwestern Medical School, Dallas) and genotyped as previously described^34^.

### *In utero* electroporation

*In utero* electroporation was performed as previously described^50^. Briefly, timed-pregnant females at E13.5 and E14.5 were deeply anaesthetised with isofluorane gas anaesthesia, the uterine horns were exposed, and 2 μl of plasmid DNA (4 μg μl^−1^) mixed with 0.05% fast green (Sigma) in PBS was manually injected through the uterus into a lateral ventricle or aqueduct using a pulled glass micropipette. For electroporation, five 50 ms pulses of 40 V with a 950 ms interval were delivered across the uterus using an electroporation generator (ECM 830, BTX, Harvard Apparatus).

### LPA injection

LPA (18:1) solution (10 mM) was dissolved in Hanks' balanced salt solution (HBSS) by sonication in a water bath for 15 min at 22 °C. A measure of 3 μl of vehicle (HBSS) or LPA solutions were injected into a lateral ventricle or aqueduct at E13.5 and E14.5. After injection, embryos were allowed to further develop and were collected after 1 day (E14.5–15.5, aqueduct), 2 days (E13.5–E15.5, aqueduct), 3 days (E14.5–E17.5, cortex) or on P0 (E13.5-P0, cortex and aqueduct).

### Histology and immunohistochemistry

Mouse brains were fixed in 4% paraformaldehyde in PBS at 4 °C for overnight, embedded in paraffin and sectioned at 7 μm in the coronal or sagittal planes. For histological analyses, sections were rehydrated and stained with hematoxylin and eosin. For immunostaining, after rehydration, sections were incubated in boiling citrate buffer (10 mM, pH6.0) for 30 min for antigen retrieval. After three washes with PBS, primary antibodies (Supplementary Methods) were incubated with 5% normal goat or donkey serum in PBS at 4 °C for overnight. Sections were then washed in PBS and incubated with the secondary antibodies. Primary antibodies information can be found in Supplementary Information. For secondary antibodies, Alexa Fluor 488-conjugated goat anti-mouse, anti-rabbit or anti-chicken, 647 conjugated goat anti-mouse (Invitrogen), Cy3-conjugated goat anti-mouse or anti-rabbit (Jackson ImmunoResearch), and biotin-conjugated goat anti-rabbit (Vector Labs) were used. Images were acquired using Axioplan 2 (Carl Zeiss) and confocal microscopes (TCS SP5 and SP8, Leica) and analysed with LAS AF (Leica) and Photoshop (Adobe).

### Western blot analysis

Aqueduct tissue was prepared for western blot by isolating the midbrain after removing the dorsal SCO at P0. For western blot analyses of LPA or HBSS cortex, the dorsal cortex was excised at E14.5, 24 h after injection of LPA or HBSS. Primary antibodies are listed in Supplementary Method and uncropped blot can be found in Supplementary Fig. 13.

### Birthdating of ependymal cells in the aqueduct

To determine the birthdates of ependymal cells, BrdU (50 mg kg^−1^) was applied to time-pregnant females through one intraperitoneal injection at E12.5, E14.5 and E16.5. Paraffin sections of the rostral aqueduct were cut and stained with antibodies for BrdU and other markers such as S100β, PTBP1 and Sox9. Double-positive cells (BrdU (+) and other marker (+)) or BrdU (+) cells in the apical lining were quantified in three sections from three WT or three *Yap* CKO embryos after image acquisition with a confocal microscope (TCS SP5 and SP8, Leica).

### Dye injection testing for blockage of CSF flow

A measure of 4 μl of 1% fast green was injected into the lateral ventricle at P0, P3 or P5 (*n*=3 for each time point) using a pulled glass micropipette. Mice were then euthanized and the brains were imaged by microscopy. The diffusion of the dye from the lateral ventricle into the fourth ventricle was determined by analysing brains 10 min post injection.

### Quantitation and statistical analyses

Three to five animals were studied for each genotype or experimental condition and cells were counted from two to three nonconsecutive sections. Statistical significance was assessed with Student's *t*-test using SigmaPlot (Systat Software, San Jose, CA).

## Additional information

**How to cite this article:** Park, R. *et al*. Yap is required for ependymal integrity and is suppressed in LPA-induced hydrocephalus. *Nat. Commun.* 7:10329 doi: 10.1038/ncomms10329 (2016).

## Supplementary Material

Supplementary Information Figures 1-13, Supplementary Methods and Supplementary Reference.
