# Supplementary material for: Yap is required for ependymal integrity and is suppressed in LPA-induced hydrocephalus
Source: Nat Commun. 2016 Jan 12;7:10329. doi: 10.1038/ncomms10329 (PMC4729961; doi:10.1038/ncomms10329)
Supplement: Supplementary Information — Figures 1-13, Supplementary Methods and Supplementary Reference. [file ncomms10329-s1.pdf]

SUPPLEMENTARY FIGURE

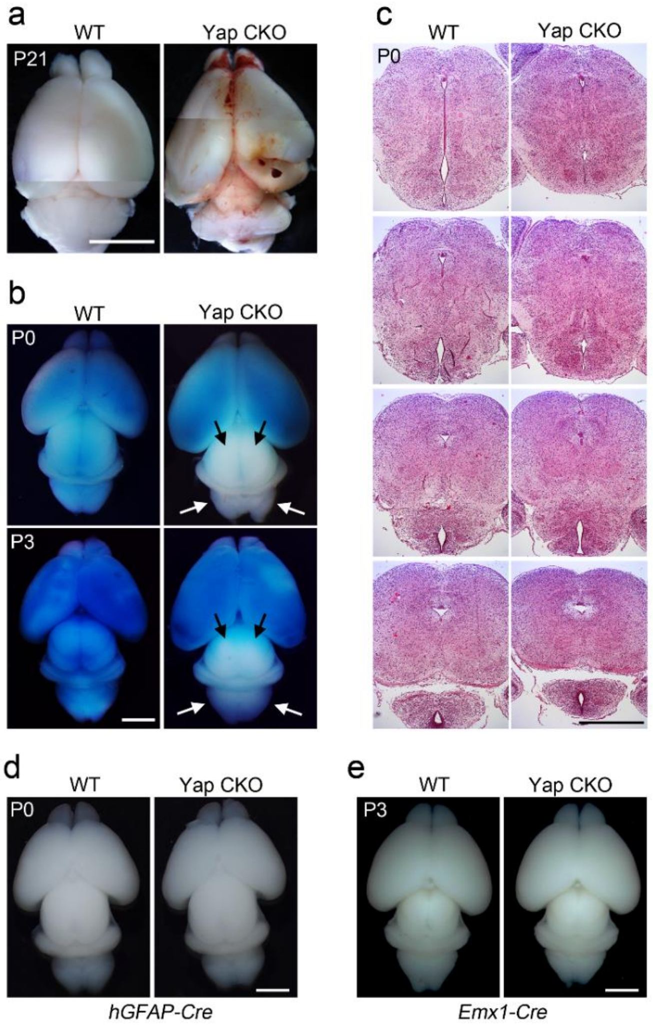

Supplementary Fig 1 *Yap* deletion generates non-communicating hydrocephalus

(**a**) At P21, a surviving *Yap* CKO shows a severe hydrocephalus phenotype. (**b**) The *Yap* CKO also shows blockage of CSF flow in the aqueduct (black arrow) and 4th ventricle (white arrow) at P0 and P3. (**c**) Low magnification pictures show the obstructed region of the aqueduct. (d, e) Hydrocephalus phenotype was not observed in animals with Yap deletion using hGFAP Cre (**d**) or Emx1Cre (**e**). Scale bars: 5mm (**a**), 1mm (**b**, **c**, **d**, **e**)

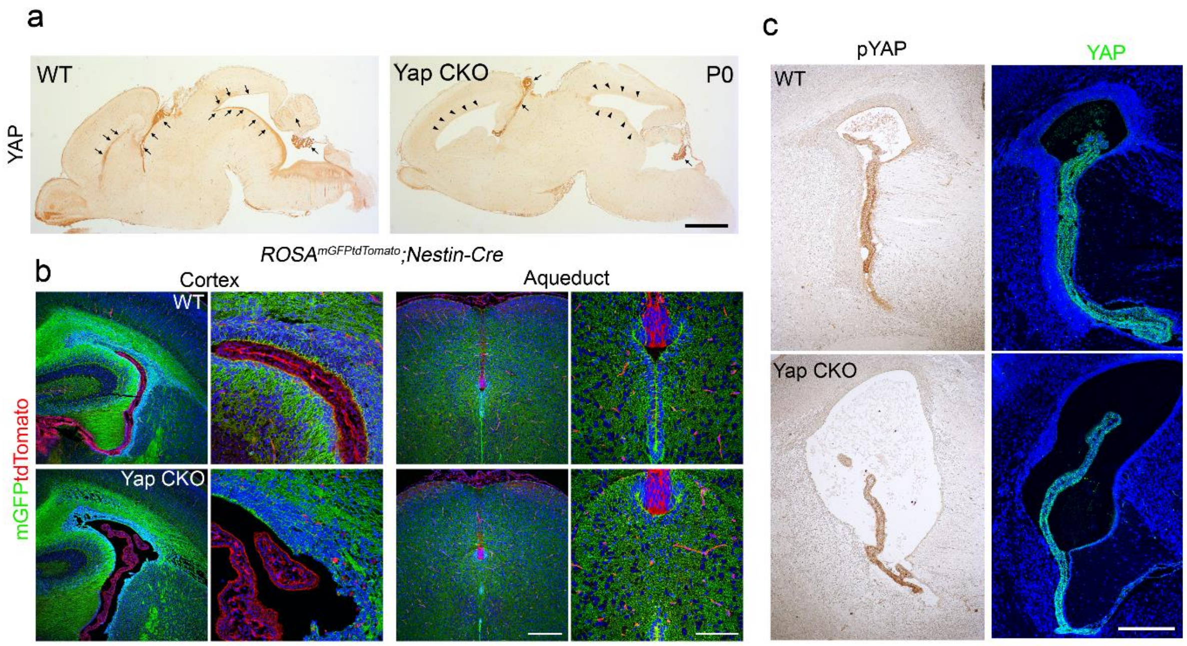

Supplementary Figure 2 Yap deletion in ventricular lining cells of aqueduct by nestin Cre

(**a**) At P0, Yap is enriched in cells in the ventricular lining of the brain (arrow), where it is largely absent in the *Yap* CKO (arrowheads). (**b**) Cre recombinase activity is not detected in SCO or choroid plexus (CP) when Nestin Cre is crossed with Rosa reporter line. Upon cre mediated recombination, GFP will be expressed, whereas tdTomato expression will mark cells which are devoid of Cre mediated recombination. (**c**) Yap expression is intact in the CP of *Yap* CKO due to the absence of *Nestin Cre* expression (arrows). Scale bars: 1mm (**a**), 300μm, 100μm (**b**), 300μm (**c**)

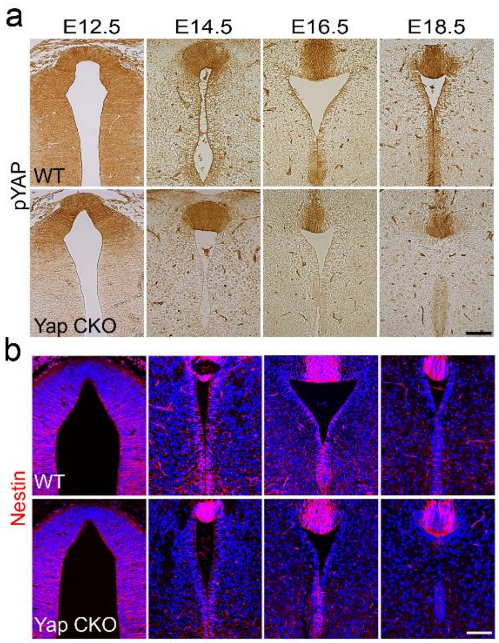

Supplementary Figure 3 Neural progenitor cells in the developing aqueduct are reduced in the *Yap* CKO

(**a**) In WT, pYap (S112) expression is found in the dorsal SCO and in proliferating cells in the ventral aqueduct at E12.5 and E14.5. It continues to be expressed in the apical lining cells at E16.5 and E18.5. In the *Yap* CKO, however, starting at E12.5, pYap is markedly reduced in the ventricular aqueduct. (**b**) Nestin expression is indistinguishable in the WT and *Yap* CKO at E12.5, but its expression is clearly less in the *Yap* CKO than in WT at E14.5. Nestin expression is decreased in the WT and *Yap* CKO at E16.5 and E18.5. Scale bars: 100μm

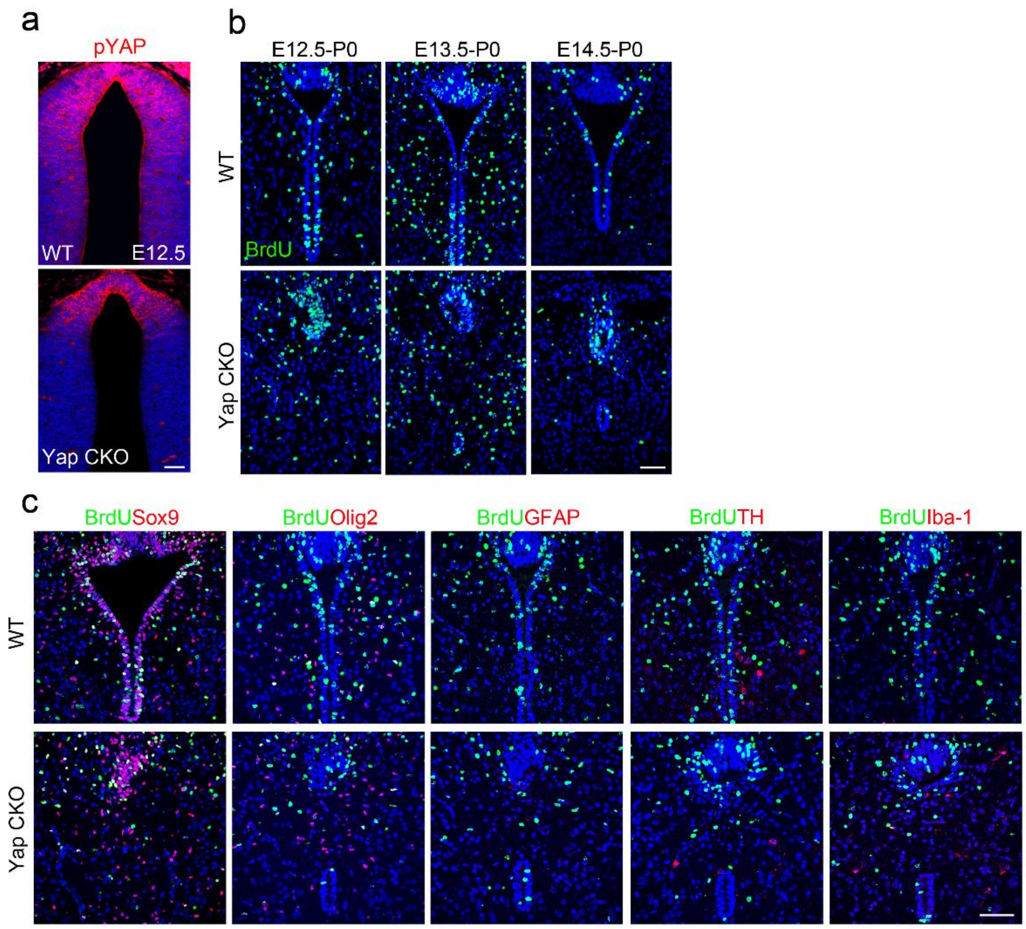

Supplementary Figure 4 Ependymal cells along the ventricular aqueduct are largely absent in the *Yap* CKO

(**a**) At E12.5, *Yap* CKO show obvious reduction of Yap expression in proliferating cells. (**b**) At P0, ependymal cells born during development (E12.5, 13.5 or E14.5) are not present in the *Yap* CKO. **(c)** At P0, cells born on E12.5, marked by BrdU expression (BrdU injection at E12.5), rarely overlap with Olig2, GFAP, TH or Iba1. Scale bars: 50µm

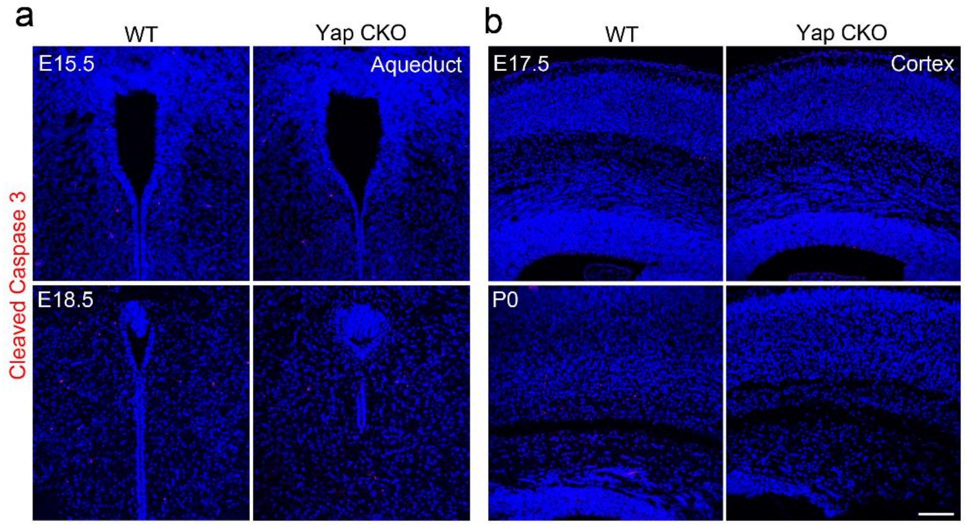

Supplementary Figure 5 Apoptotic cell death is not distinctively changed in the *Yap* CKO

(**a**) In *Yap* CKO, dying cells labeled by cleaved caspase 3 expression in the aqueduct at E15.5 and E18.5 are indistinguishable from those of WT. (**b**) In the developing cortex, CC3 labeled cells in the *Yap* CKO do not differ from WT at E17.5 or P0. Scale bars: 100μm

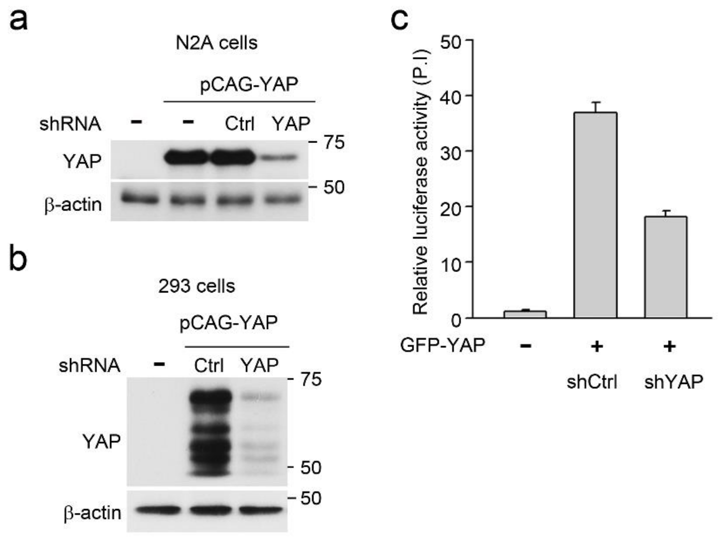

Supplementary Figure 6 *Yap* shRNA decreases Yap expression and transcriptional activity

(**a, b**) Concurrent transfection of shRNA construct (details in Materials and Methods) and *Yap* overexpression driven by CAG promoter show that *Yap* shRNA efficiently reduces Yap expression in N2A cells (**a**) and H293 cells (**b**). (**c**) Luciferase assay of Yap-TEAD reporter construct reveals reduction of transcription activation by Yap when Yap shRNA is concomitantly expressed in MCF7 cells.

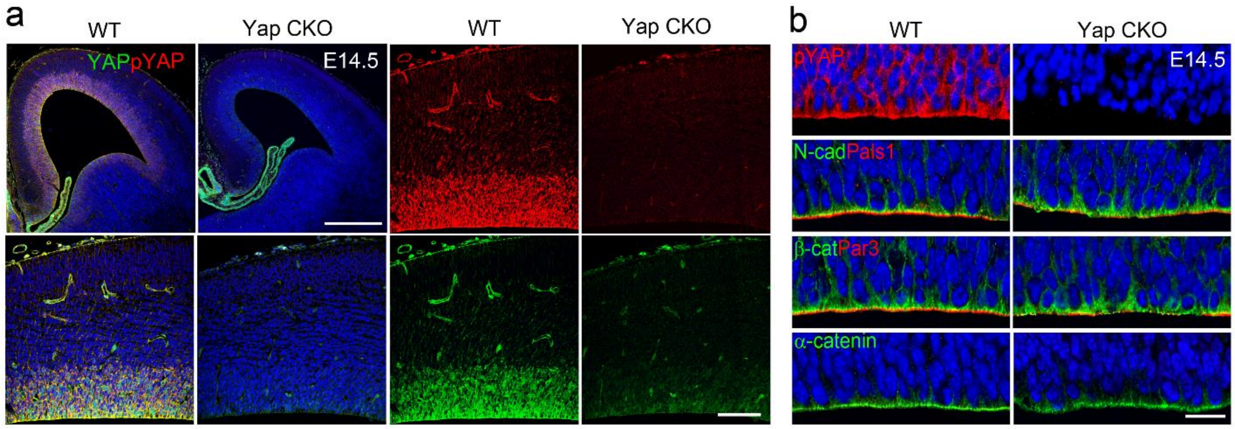

Supplementary Figure 7 Yap is required for normal localization of junction proteins in cortical progenitors

(a) Both total Yap and pYap expression in progenitors are substantially diminished in the *Yap* CKO at E14.5. (**b**) Expression of AJ proteins such as N-Cadherin, α-Catenin and β-Catenin in the *Yap* CKO is slightly disorganized and less dense than normal, and concentration of apical polarity complex proteins along the ventricular surface is decreased at E14.5. Scale bars: 500μm, 100μm (**a**), 20μm (**b**)

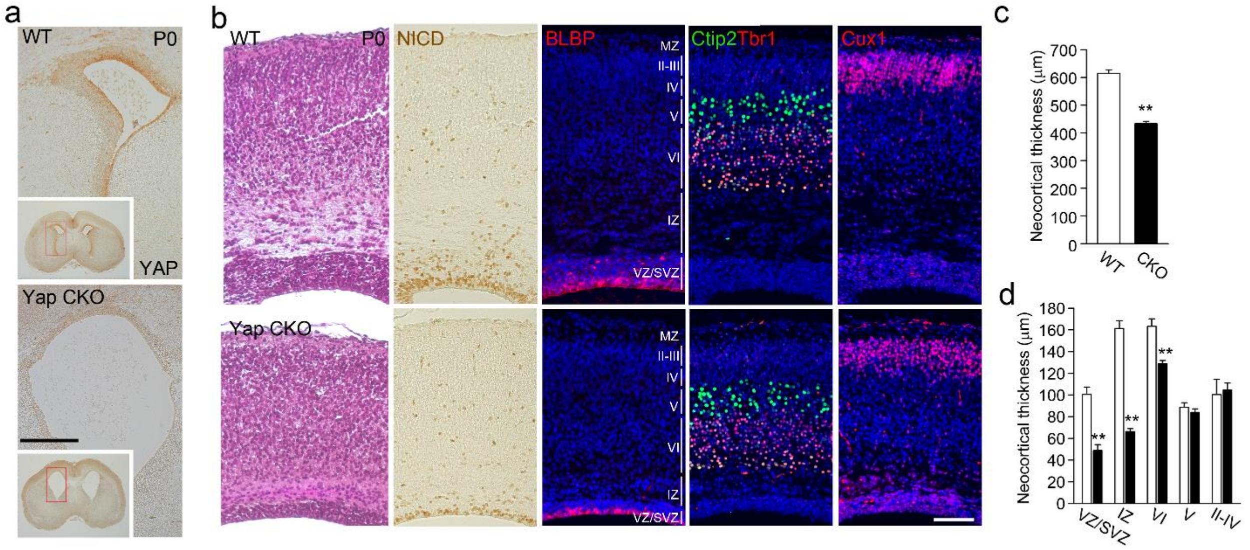

Supplementary Figure 8 Neurogenesis proceeds relatively normally in the *Yap* CKO

(**a**) Yap expression in the cortex at P0 is substantially reduced in the *Yap* CKO (**b, c, d**) Histology of cortex demonstrates reduced thickness in the Yap CKO, especially IZ and VZ (**c, d**). VZ cells marked by NICD or BLBP are reduced in thickness. Layer 6, including Tbr1 (+) layer 6 neurons, is also thinner in Yap CKO than WT (**d**). However, Ctip2 (+) layer 5 and Cux1 (+) layer 2-4 neurons are ordered correctly in the compressed cortex, and thickness was not affected by the increased CSF pressure (**d**). Two-tailed unpaired *t*-test reveals statistical significance (***P*<0.01). Scale bars: 300μm (**a**), 100μm (**b**)

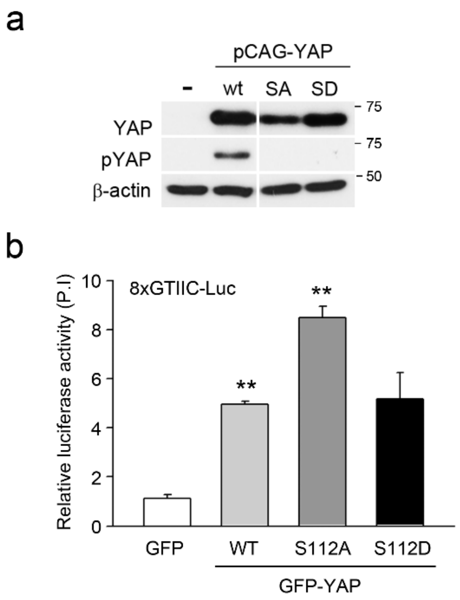

Supplementary Figure 9 *Yap* (S112) mutant constructs have different effects on transcriptional activation

(**a**) Overexpression of Yap mutant constructs at Serine 112 phosphorylation site in N2A cells demonstrates that phosphorylation site at S112 mutants cannot be detected by Phosphor site specific antibody. (**b**) Phosphodefective mutant (S112 to A) elevates transcription most effectively; Phosphomimetic mutant and Yap WT mutant have comparable transcriptional activation activity monitored by Yap-TEAD reporter Luciferase assay in MCF7 cells. Two-tailed unpaired t-test reveals statistical significance (**P<0.01).

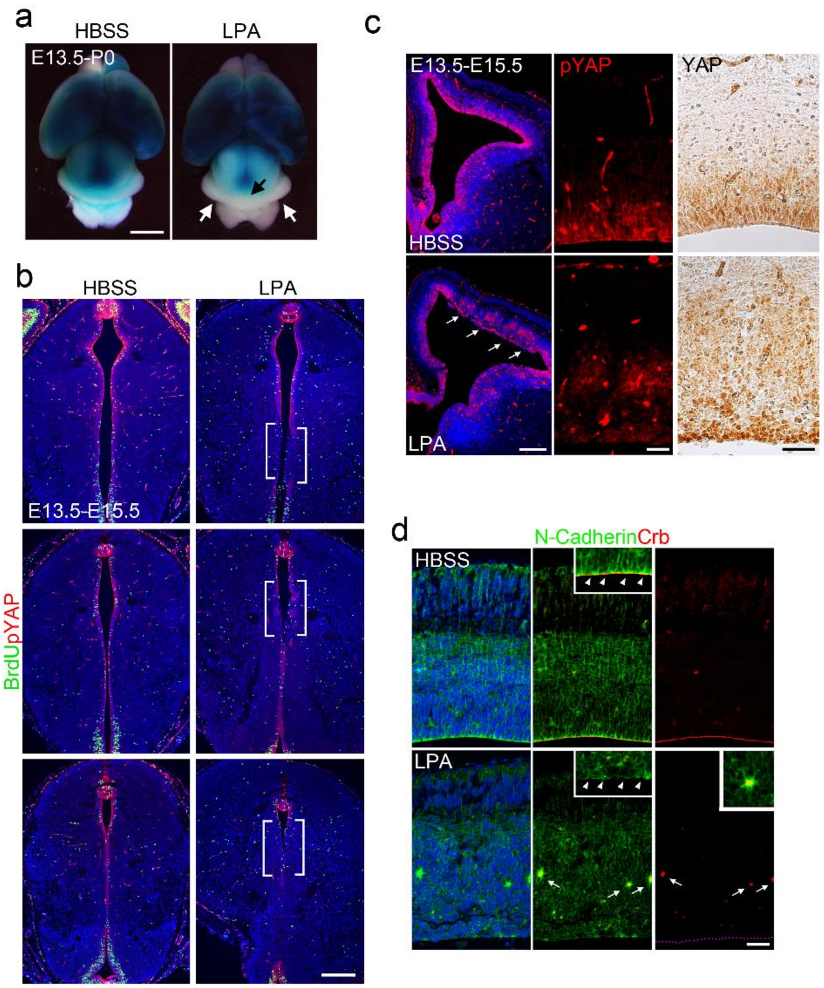

Supplementary Figure 10 LPA mediated hydrocephalus is similar to that of *Yap* CKO

(**a**) LPA injection into a lateral ventricle at E13.5 induces hydrocephalus and aqueduct stenosis, which blocks dye flow from the lateral ventricle to the 4th ventricle (arrows) at P0. (**b**) Two days after LPA injection, Yap distribution is already decreased in the ventricular surface of the aqueduct (brackets). (**c**) LPA application induces loss of cytoplasmic and junctional Yap, but concentrated nuclear Yap is detected in the apical surface of cortex (arrows). (**d**) LPA injection disrupts cell and tissue polarity, as shown by abnormal distribution of AJ proteins such as N-Cadherin and apical polarity complex proteins such as Crb in rosettes (arrows), and loss of their

localization at the apical surface (arrowheads). Scale bars: 1mm (**a**), 200μm (**b**), 300μm and 50μm (**c**), 50μm (**d**)

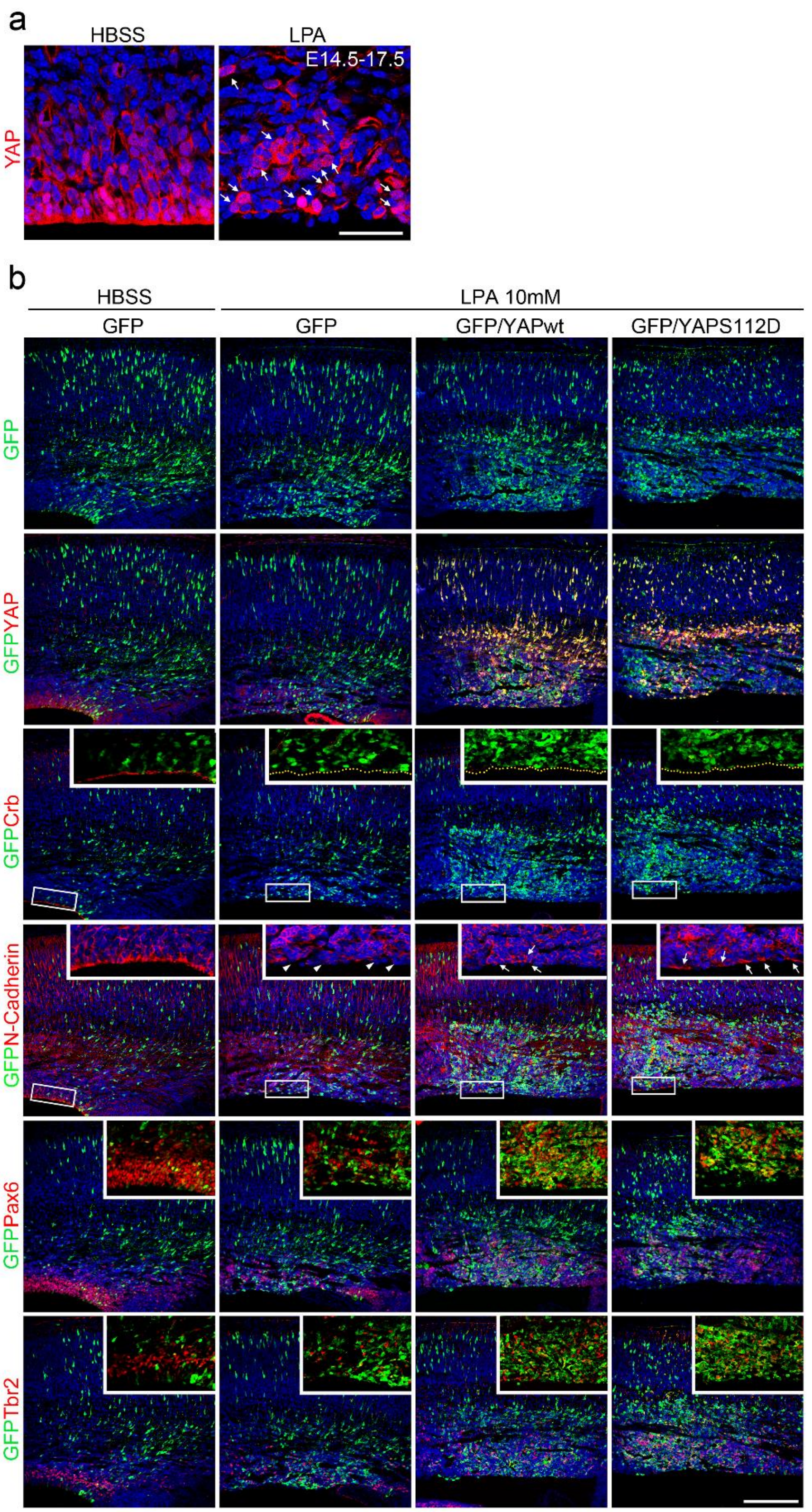

a
HBSS
LPA
E14.5-17.5
YAP
b
HBSS
LPA 10mM
GFP
GFP
GFP/YAPwt
GFP/YAPS112D
GFP
GFPYAP
GFPCrb
GFPN-Cadherin
GFPPax6
GFPTbr2

Supplementary Figure 11 Yap overexpression partially restores apical integrity altered by LPA

(**a**) Three days after LPA injection at E14.5, Yap is profoundly reduced in the cytoplasm and junction but retained in the nucleus of some cells (arrows). (**b**) Site directed mutagenesis generates phospho-defective Yap (S112A) and phosphomimetic Yap (S112D) constructs. Yap WT and mutant proteins are detected by Yap antibody, but neither is recognized by phospho-specific antibody at S112. (**c**) Concurrent LPA injection and overexpression of Yap or phosphomimetic Yap (S112D) partially restores N-Cadherin at the apical surface. Crb localization in the apical surface is not restored, and apical progenitors (Pax6+) and basal progenitors (Tbr2+) remain scattered despite Yap WT or mutant protein overexpression. Scale bars: 50μm (**a**), 200μm (**b**)

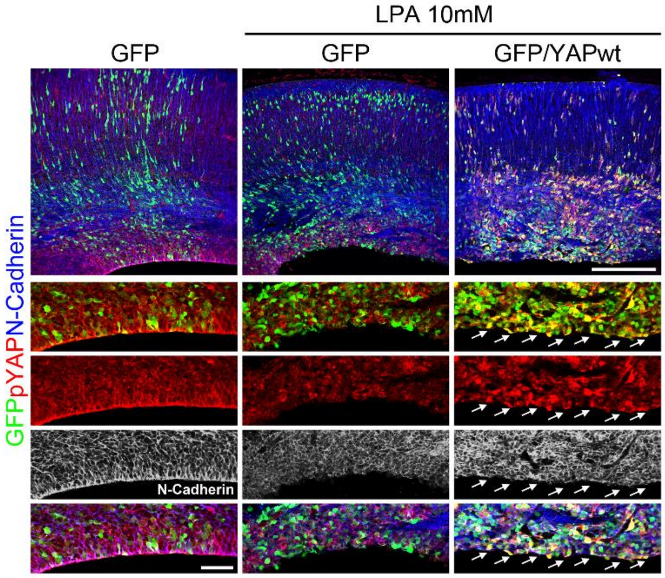

Supplementary Figure 12 Yap overexpression leads to the accumulation of pYap at the apical surface

N-Cadherin extensively overlaps with pYap in the cell junctions and apical surface of the control. Both N-cadherin and pYap are substantially diminished when LPA is administered. Concurrent LPA injection and overexpression of Yap through electroporation shows accumulation of pYap at the apical surface and cell junctions. Yap overexpressing cells labelled by GFP show restoration of N-Cadherin localization at the apical surface and cell junction where pYap is highly enriched (arrows). Scale bars: 200µm and 50µm

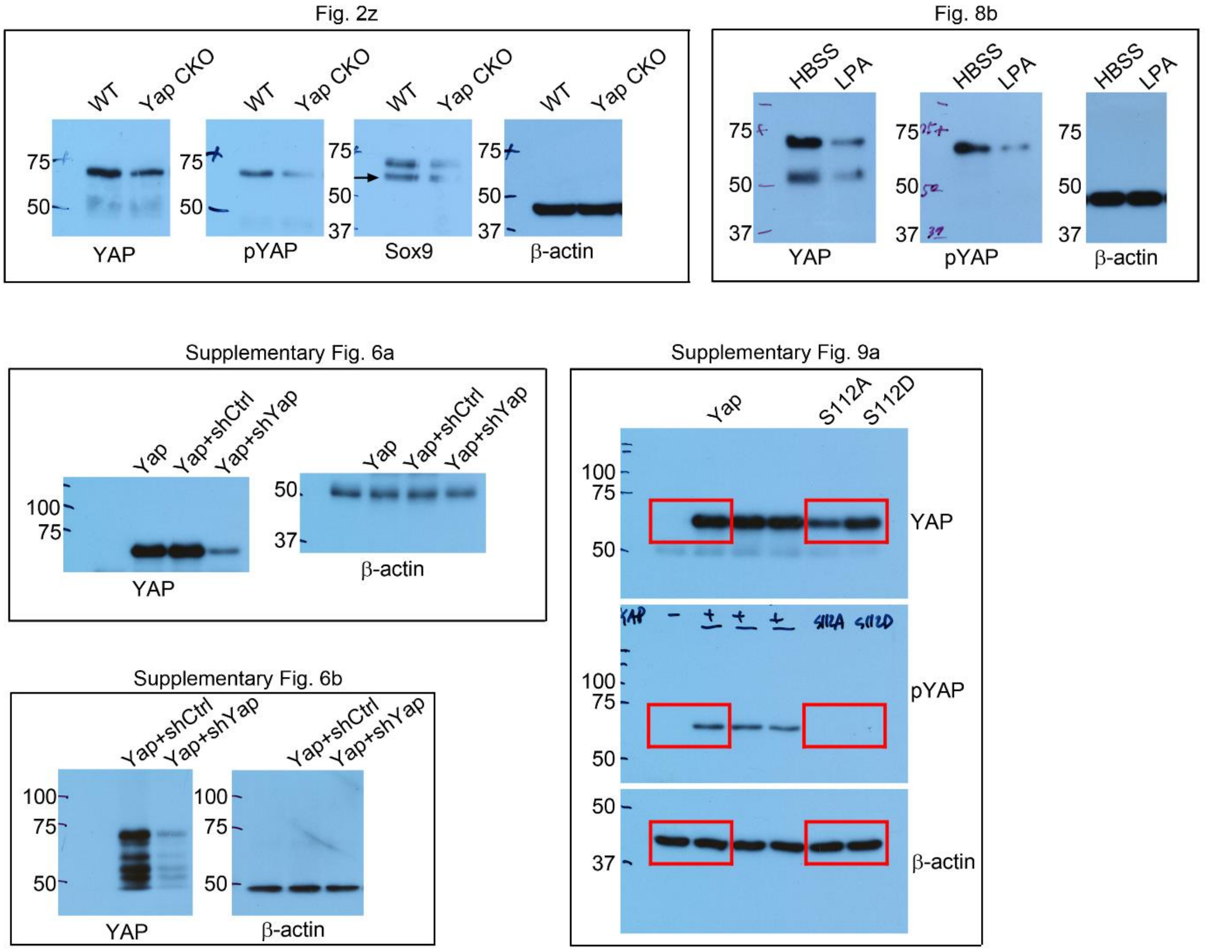

Supplementary Fig 13 Uncropped western blots of Fig 2, 8, and supplementary fig 6, 9

SUPPLEMENTARY METHODS

DNA constructs

The open reading frames of *Yap* and *Yap* mutant constructs, including S112A and S112D, were cloned by a PCR-based method using full length mouse *Yap* obtained from MGC clone (Id:4239820, Thermo Scientific) as a template. To generate N-terminal GFP tagged Yap, Yap full length was inserted in frame to GFP coding sequence. Initially, the PCR product was cloned into *T-easy* vector and the *NotI* digested insert including *Yap, Yap* mutants *or GFP-Yap* was cloned into the *NotI* site of the *pCAGEN* vector. For shRNA construct, 'CGGTTGAAACAACAGGAATTA' sequence[1] was cloned into *pLlox3.7* vector.

Antibodies

Primary antibodies: YAP (ab56701, Abcam, 1:200), YAP (#4912, Cell Signaling, 1:100), pYAP (#4911, Cell Signaling, 1:100), BrdU (347580, BD, 1:50), BrdU (ab6326, Abcam, 1:200), pH3 (06570, Millipore, 1:200), Pax6 (RBP-278P, Covance, 1:200), Tbr1 (AB2261, Millipore, 1:1000), Pals1 (07-708, Millipore, 1:200), pan-Crb2 (Synthesized, 1:500), N-Cadherin (610920, BD, 1:200), α-Catenin (610193, BD, 1:200), β-Catenin (610153, BD, 1:200), Par3 (07330, Millipore, 1:200), aPKC (610207, BD, 1:200), PTBP1 (sc-16547, Santa Cruz, 1:200), S100β (NB110-57478, Novus, 1:200), Adenylyl Cyclase III (sc-588, Santa Cruz, 1:200), BLBP (ABN14, Millipore, 1:200), NICD (#4147, Cell Signaling, 1:200), Sox9 (AB5535, Millipore, 1:200), Cux1 (sc-13024, Santa Cruz, 1:200), Ctip2 (ab28448, Abcam, 1:200), GFP (GFP-1020, Aves, 1:200), GFP (ab13970, Abcam, 1:200), NeuN (MAB377, Millipore, 1:200), βIII-tubulin (MMS-435P, Covance, 1:500), Cleaved Caspase 3 (#9661, Cell Signaling, 1:200), Nestin

(DSHB, 1:200), Olig2 (AB9610, Millipore, 1:200), GFAP (RB-087, Thermo, 1:200), Tyrosine Hydroxylase (ab152, Millipore, 1:200), Iba-1 (#019-19741, Wako, 1:200), β-actin (sc-47778, Santa Cruz, 1:1000).

Western blot analysis

For evaluating plasmid constructs (*pCAG-GFP, pCAG-GFP-Yap, pCAG-GFP-Yap S112A, pCAG-GFP-Yap S112D, pLlox3.7-shControl* or *shYap*), constructs were transfected to N2A or 293 cells; cells were harvested after 24 hrs and used for Western blots. Primary antibodies are YAP (ab56701, Abcam, 1:1000), pYAP (#4911, Cell Signaling, 1:1000), Sox9 (AB5535, Millipore, 1:1000), and β-actin (sc-47778, Santa Cruz, 1:2000).

BrdU labeling

Proliferating cells in the S-phase were labeled by BrdU injection (50 mg/kg), and embryos were harvested after 30 min. For cell cycle exit analyses, BrdU injection was performed at E13.5, and embryos were harvested at E14.5. For BrdU birthdating, BrdU was injected to pregnant dams at E12.5, E14.5 or E16.5, and tissue was harvested at P0. Two nonconsecutive sections were used for BrdU staining, and BrdU (+) cells in the apical lining of the aqueduct of WT and *Yap* CKO mice (N=3, each group) were counted manually from 40X images taken with a Zeiss Axiopot. For fate analyses of BrdU (+) cells after BrdU injection at E12.5, confocal images (40x) were taken from the rostral aqueduct of WT and CKO mice (N=3, each group), and BrdU (+) or BrdU (+) and Sox9 (+) or NeuN (+) cells were counted manually. We compared the fraction of BrdU (+) and Sox9 (+) or NeuN (+) cells among total BrdU (+) cells in a given image.

Luciferase Assay

pGL3 or *pGL3-8xGTIIC* vector (Addgene) and *pCMV-Renilla* as an internal control were used as reporter constructs to monitor Luciferase activity. *pCAG-GFP*, *pCAG-GFP-Yap*, *pCAG-GFP-Yap S112A*, *pCAG-GFP-Yap S112D*, *pLlox3.7-shControl* or s*hYap* were transfected along with the Luciferase reporter constructs into MCF7 cells. 24 hrs after transfection of these constructs, firefly luciferase expression was assessed by using the Dual-Glo Luciferase Assay System (Promega). Luciferase substrate signals were read with the Orion L Microplate Luminometer (Titertek). Triplicates of independent transfection experiments were performed and statistical significance was evaluated with Student's t-test.

SUPPLEMENTARY REFERENCE

1. Zhang, H., Pasolli, H.A. & Fuchs, E. Yes-associated protein (YAP) transcriptional coactivator functions in balancing growth and differentiation in skin. *Proceedings of the National Academy of Sciences of the United States of America* **108**, 2270-5 (2011).
